# Using proteolysis-targeting chimera technology to reduce navitoclax platelet toxicity and improve its senolytic activity

**DOI:** 10.1038/s41467-020-15838-0

**Published:** 2020-04-24

**Authors:** Yonghan He, Xuan Zhang, Jianhui Chang, Ha-Neui Kim, Peiyi Zhang, Yingying Wang, Sajid Khan, Xingui Liu, Xin Zhang, Dongwen Lv, Lin Song, Wen Li, Dinesh Thummuri, Yaxia Yuan, Janet S. Wiegand, Yuma T. Ortiz, Vivekananda Budamagunta, Jennifer H. Elisseeff, Judith Campisi, Maria Almeida, Guangrong Zheng, Daohong Zhou

**Affiliations:** 1 Department of Pharmacodynamics, College of Pharmacy, University of Florida, Gainesville, FL USA; 2 Department of Medicinal Chemistry, College of Pharmacy, University of Florida, Gainesville, FL USA; 3 Department of Pharmaceutical Sciences, University of Arkansas for Medical Sciences, Little Rock, AR USA; 4 Division of Endocrinology and Metabolism, Center for Osteoporosis and Metabolic Bone Diseases, University of Arkansas for Medical Sciences, Little Rock, AR USA; 5 Translational Tissue Engineering Center, Wilmer Eye Institute, Johns Hopkins School of Medicine, Baltimore, MD USA; 6 Buck Institute for Research on Aging, Novato, CA USA; 7 Lawrence Berkeley National Laboratory, Berkeley, CA USA

**Keywords:** Drug discovery, Platelets, Geriatrics

## Abstract

Small molecules that selectively kill senescent cells (SCs), termed senolytics, have the potential to prevent and treat various age-related diseases and extend healthspan. The use of Bcl-xl inhibitors as senolytics is largely limited by their on-target and dose-limiting platelet toxicity. Here, we report the use of proteolysis-targeting chimera (PROTAC) technology to reduce the platelet toxicity of navitoclax (also known as ABT263), a Bcl-2 and Bcl-xl dual inhibitor, by converting it into PZ15227 (PZ), a Bcl-xl PROTAC, which targets Bcl-xl to the cereblon (CRBN) E3 ligase for degradation. Compared to ABT263, PZ is less toxic to platelets, but equally or slightly more potent against SCs because CRBN is poorly expressed in platelets. PZ effectively clears SCs and rejuvenates tissue stem and progenitor cells in naturally aged mice without causing severe thrombocytopenia. With further improvement, Bcl-xl PROTACs have the potential to become safer and more potent senolytic agents than Bcl-xl inhibitors.

## Abstract (web summary)

Senolytics have the potential to extend healthspan by selectively killing senescent cells (SCs), but senolytics that target Bcl-xl may cause platelet toxicity. Here, the authors generated a Bcl-xl proteolysis-targeting chimera (PROTAC) senolytic, which effectively clears SCs and rejuvenates tissue stem and progenitor cells in naturally aged mice without causing severe thrombocytopenia.

## Introduction

Senescent cells (SCs) have recently emerged as therapeutic targets for age-related diseases. SCs accumulate with age and as a result of the exposure to a variety of stressors including radiation. They play a causal role in many age-related diseases (such as neurodegenerative and cardiovascular diseases, osteoarthritis, and cancer) via expression of the senescence-associated secretory phenotype (SASP) to secret various inflammatory mediators and proteases^1–7^. Small molecules that can selectively kill SCs, termed senolytics, have the potential to prevent and treat a growing number of age-related diseases and extend healthspan^2–4^. To date, several classes of senolytics have been identified. These agents include natural compounds such as quercetin^8,9^, fisetin^10,11^, piperlongumine^12,13^, and curcumin analogs^14^; and targeted therapeutics such as dasatinib (a non-specific tyrosine kinase inhibitor)^8,15^, inhibitors of the anti-apoptotic Bcl-2 family proteins^16–18^, an inhibitor of HSP90 and a histone deacetylase^19^, UBX101 (an inhibitor of the MDM2/p53 protein interaction)^20^; and a modified FOXO4-p53 interfering peptide^21^. In addition, cytotoxic agents encapsulated with β(1,4)‐galacto‐oligosaccharides have also been used to target SCs that have high levels of lysosomal β‐galactosidase activity^22^.

While most natural senolytics have the advantages of low toxicity, they are usually less potent than targeted senolytics and must be combined with other agents to be effective in clearing SCs^8^. In addition, the mechanisms of action of most natural senolytics are not well defined nor have their molecular targets been identified and characterized, making it difficult to rationally modify them to improve their senolytic activity. By contrast, almost all the targeted senolytics are repurposed anticancer agents except the FOXO4-p53 interfering peptide^2–4^. The repurposed senolytics usually have on-target and/or off-target toxicities. These toxicities limit their clinical use as anti-aging agents because older people are more susceptible to adverse drug effects than younger individuals and less tolerant to cancer drug toxicity. Therefore, strategies to reduce on-target and/or off-target toxicity of known targeted senolytics are urgently needed.

ABT263 (also known as navitoclax), a Bcl-2 and Bcl-xl dual inhibitor^23,24^, is one of the most potent and broad-spectrum senolytic agents identified to date^16–18^. Bcl-xl inhibition with ABT263 and other small molecular inhibitors induces platelet apoptosis and results in severe thrombocytopenia, which prevents the use of ABT263 and other Bcl-xl specific inhibitors in the clinic—even for cancer patients—because platelets solely depend on Bcl-xl for survival^24–27^. By contrast, Bcl-2 is dispensable for thrombopoiesis and platelet survival in mice and humans^28^ and inhibition of Bcl-2 with ABT199 (also known as venetoclax) does not induce thrombocytopenia^27^. We hypothesize that we can reduce ABT263 on-target toxicity and generate a safer senolytic agent by converting ABT263 into a platelet-sparing Bcl-xl proteolysis-targeting chimera (PROTAC).

PROTACs are bivalent small molecules containing a ligand that recognizes a target protein linked to another ligand that recruits a specific E3 ubiquitin ligase^29,30^. PROTAC binding induces proximity-induced ubiquitination of the target protein and its subsequent degradation by proteasomes. Thus, PROTACs act catalytically to induce protein degradation in a sub-stoichiometric manner. Since their effect is not limited by equilibrium occupancy, it results in less drug exposure and reduced toxicity compared with traditional inhibitors. Because of their improved and prolonged activity profile, they are increasingly used to develop more effective antitumor agents and other therapeutics^31–33^. Importantly, because PROTACs rely on E3 ligases to induce protein degradation, it is possible to achieve cell/tissue selectivity, even when the target proteins are ubiquitously expressed as long as they target the proteins to an E3 ligase that is cell- or tissue-specific.

Here, we report the use of PROTAC technology to reduce ABT263 on-target toxicity by converting ABT263 into PZ15227 (PZ), a Bcl-xl specific PROTAC (Bcl-xl-P), which targets Bcl-xl to the E3 ligase cereblon (CRBN) that is poorly expressed in platelets^34,35^. We find that PZ is less toxic to platelets but equally or slightly more potent against SCs compared with ABT263. These findings provide an approach to reduce on-target toxicity of toxic senolytic agents. With further improvement, Bcl-xl-Ps have the potential to be developed into safer and more effective senolytics than ABT263.

## Results

### PZ selectively kills SCs with reduced platelet toxicity

**Fig. 1:**
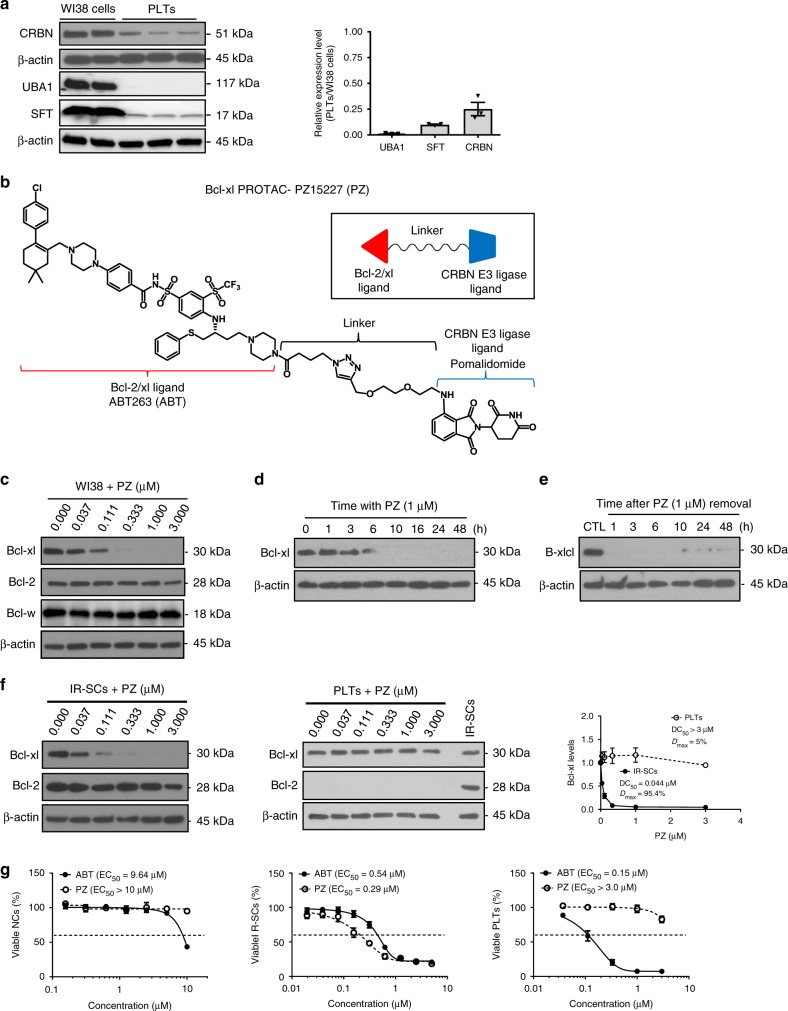
**PZ15227 (PZ) is a more potent senolytic agent but less toxic to platelets than ABT263**. **a** Human platelets (PLTs) express significant lower levels of E3 ligase cereblon (CRBN), ubiquitin activating enzyme E1 (UBA1) and ubiquitin-conjugating enzyme E2 (SFT) than WI38 cells. The expression of ubiquitin enzymes (E1, E2, and E3) was normalized to β-actin and presented as the fold change relative to WI38 cells. The data presented in the bar graph are mean ± SEM (*n* = 3 different samples). **b** Molecular structure of PZ showing a Bcl-2/xl ligand linked to a CRBN ligand via an optimized linker. **c** PZ selectively induced degradation of Bcl-xl, but not Bcl-2 and Bcl-w, in a dose-dependent manner in WI38 cells after PZ treatment for 16 h. **d** PZ-induced Bcl-xl degradation in WI38 cells in a time-dependent manner. **e** PZ-induced Bcl-xl degradation persisted up to 48 h upon the removal of PZ after 16 h PZ treatment. **f**. PZ-induced degradation of Bcl-xl, but not Bcl-2, in WI38 senescent cells (SCs) induced by ionizing radiation (IR-SCs) (left), but not in human PLTs (middle), in a dose-dependent manner after they were treated with increasing concentrations of PZ for 16 h. Densitometric analyses of Bcl-xl expression in IR-SCs and PLTs are presented as mean ± SEM (*n* = 4 independent assays) (right). DC_50_, drug concentration causing 50% Bcl-xl degradation; *D*_max_, the maximum level of Bcl-xl degradation. **c**–**f** Similar results were got in at least two independent experiments. **g** PZ is more potent against IR-SCs but less toxic to WI38 non-senescent cells (NCs) and human PLTs than ABT263. The viability of NCs (left), IR-SCs (middle) and human PLTs (right) was determined 72 h after PZ or ABT263 treatment. EC_50_, half-maximal effective concentration. The data presented in Fig. 1g are mean ± SEM (*n* = 3 independent assays). Source data are provided as a Source Data file.

By analyzing previously published human platelet RNA sequencing data, we found that the CRBN E3 ligase and its associated E1 (UBA1) and E2 (SFT) enzymes are poorly expressed in human platelets^34,35^. We confirmed this finding by western blot analysis and found that human platelets express significantly lower levels of CRBN, UBA1, and SFT than other cells including WI38 human fibroblast cells (WI38 cells) (Fig. 1a). Based on this finding, we rationally designed and synthesized a series of Bcl-xl-Ps that target Bcl-xl to CRBN for degradation by tethering a Bcl-2/Bcl-xl binding moiety derived from ABT263 to a CRBN binding moiety pomalidomide via a linker (Fig. 1b). Among these Bcl-xl-Ps, PZ was selected as a lead for further evaluation because it has high Bcl-xl binding affinity (Supplementary Fig. 1a) and could potently induce Bcl-xl degradation in WI38 non-senescent cells (NCs) with the half-maximal degradation concentration (DC_50_) at 46 nM and maximum degradation (*D*_max_) of 96.2% (Fig. 1c and Supplementary Fig. 1b). The induction of Bcl-xl degradation by PZ was rapid (Fig. 1d) and long lasting (Fig. 1e). However, it had no significant effect on Bcl-2 and Bcl-w protein levels, probably due to a significantly reduced binding to Bcl-2 and Bcl-w (Fig. 1c and Supplementary Fig. 1a, c, d); nor did it affect the expression of *Bcl2l1* (*Bcl-xl*) mRNA in WI38 NCs (Supplementary Fig. 1e). These findings suggest that PZ is a specific Bcl-xl-P. This is further supported by the observations that PZ could selectively induce Bcl-xl but not Bcl-2 poly-ubiquitination and had no significant effect on the expression of the CRBN native substrate, glutamine synthetase (GS)^36^, and its neosubstrates recruited by thalidomide and its analogues including CK1α^37^, IKZF1, and IKZF3 (ref. ^38^) (Supplementary Fig. 1 f, g). More importantly, PZ had similar potency in degrading Bcl-xl in WI38 SCs induced by ionizing radiation (IR-SCs) (Fig. 1f, left panel) as in WI38 NCs (Fig. 1c), but had no effect on the levels of Bcl-xl in human platelets (Fig. 1f, middle panel).

Because both SCs and platelets, but not NCs, are primarily dependent on Bcl-xl for survival^16–18,24–27^ (Supplementary Fig. 1h), we evaluated the effects of PZ compared with ABT263 on the viability of WI38 NCs (Fig. 1g, left panel), IR-SCs (Fig. 1g, middle panel), and platelets (Fig. 1g, right panel). As reported, ABT263 was highly toxic to both WI38 IR-SCs (Fig. 1g, middle panel) and platelets^16–18,24–27^ (Fig. 1g, right panel; Supplementary Fig. 2). By contrast, PZ exhibited a slightly improved potency in inducing apoptosis in WI38 IR-SCs compared with ABT263 (Fig. 1g, middle panel; Supplementary Fig. 3), but had minimal effect on the viability of platelets even at 3 µM (Fig. 1g, right panel; Supplementary Fig. 2) and was less toxic to WI38 NCs (Fig. 1g, left panel; Supplementary Fig. 3), resulting in a more than 37-fold increase in platelet toxicity index (i.e. ratio of EC_50-PTLs_/EC_50-IR-SCs_) between PZ and ABT263. This improved senolytic activity of PZ was also observed in WI38 SCs induced by extensive replication (RE-SCs) and in SCs derived from renal epithelial cells (REC) and pre-adipocytes (PAC) induced by irradiation (Supplementary Fig. 4). However, the senolytic activity of PZ was not significantly different from that of ABT263 in WI38 SCs induced by ectopic transfection of *Ras* oncogene (Ras-SCs) and IMR90 SCs induced by irradiation (Supplementary Fig. 4), suggesting that there are some differences among SCs derived from different cellular origins and induced by different stressors in their response to PZ and ABT263. Importantly, PZ is also substantially less toxic to REC-NCs and PAC-NCs than ABT263. These findings confirm that PZ is a potent broad-spectrum senolytic agent that has a slightly improved senolytic activity against the majority of SCs studied, yet low toxicity to platelets and NCs compared with ABT263.

### Effects of PZ depend on CRBN and proteasome activity

**Fig. 2:**
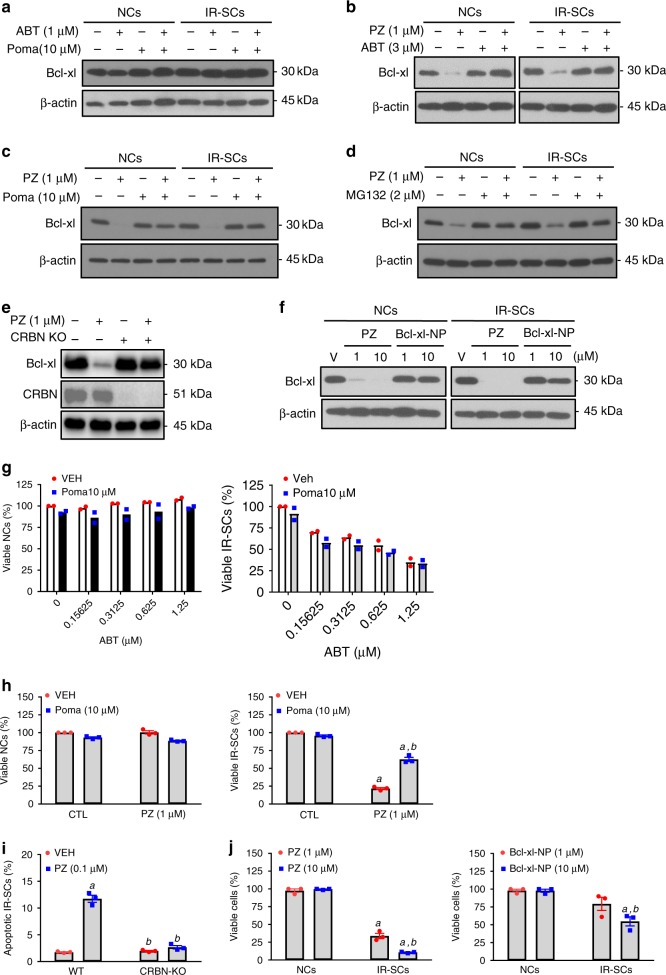
**PZ induces Bcl-xl degradation depending on the CRBN E3 ligase and proteasomes**. **a** No effect of ABT263 and/or the CRBN ligand pomalidomide (Poma) on Bcl-xl in WI38 NCs and IR-SCs. **b-d** ABT263, Poma and MG132 (a proteasome inhibitor) pretreatment blocked the degradation of Bcl-xl by PZ in WI38 NCs and IR-SCs, respectively. **e** CRBN knockout (KO) blocked Bcl-xl degradation by PZ in WI38 IR-SCs. **f** PZ, but not Bcl-xl-NP (an inactive form of PZ that cannot bind to CRBN), induced Bcl-xl degradation in NCs and IR-SCs. **a**–**f** Similar results were got in at least two independent experiments. **g** ABT263 and/or Poma did not induce cell death in NCs (left), while ABT263, but not Poma, induced cell death in IR-SCs (right). The data presented are mean value (*n* = 2 independent experiments). **h** Poma pretreatment blocked the effects of PZ on cell viability. Cells were pretreated with Poma for 1 h prior to PZ treatment, and cell viability in NCs (left) and IR-SCs (right) was measured 72 h after PZ treatment. The data presented are mean ± SEM (*n* = 3 independent experiments). *a*, *b* *p* < 0.0001 vs. control (CTL) and vehicle (VEH)-treated IR-SCs, respectively. **i** Knockout CRBN blocked the effects of PZ on cell viability. Cell viability was measured in wild-type (WT) and CRBN knockout (KO) IR-SCs 72 h after PZ treatment. The data presented are mean ± SEM (*n* = 3 independent experiments). *a*, *b* *p* < 0.0001 vs. VEH- and PZ-treated WT IR-SCs, respectively. **j** PZ (left), but not Bcl-xl-NP (right), effectively killed IR-SCs. The data presented are mean ± SEM (*n* = 3 independent experiments). *a* *p* < 0.0001, and *b* *p* = 0.0004 vs. NCs and IR-SCs treated with 1 μM PZ (left). *a* *p* = 0.0045 and *b* *p* = 0.0337 vs. NCs and IR-SCs treated with 1 μM Bcl-xl-NP (right), respectively. **a**–**f** Cells were pretreated with or without indicated compounds for 1 h and treated with PZ for 16 h. All statistical analyses presented were done by two-way ANOVA with Tukey’s post-hoc test. Source data are provided as a Source Data file.

**Fig. 3:**
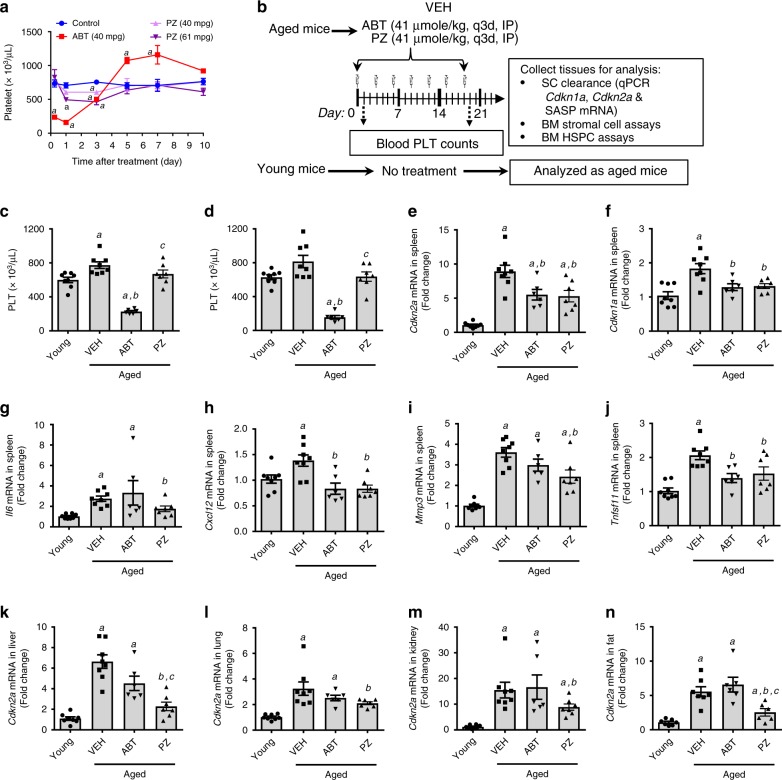
**PZ effectively clears SCs without causing thrombocytopenia in naturally aged mice**. **a** Mice received an IP injection of vehicle (VEH), ABT263 (ABT) or PZ, and platelets (PLTs) were numerated at 6 h, day 1, 3, 5, 7, and 10 after the injection. Data are represented as mean ± SEM (*n* = 3 mice/group). a *p* < 0.05 vs. control, determined by one-way ANOVA with Tukey’s post-hoc tests. **b** Illustration of the experiment design. Naturally aged mice (Aged) were given 41 μmol/kg of PZ or ABT263 by IP injection every 3 days (q3d) for a total of 7 injections and euthanized 6 days after the last treatment to harvest various tissues and cells for analyses. Control untreated young mice (Young) mice were analyzed as aged mice without any treatment. Blood PLTs counts in Young and naturally aged mice were measured 1 day after receiving the 1st (**c**) and 7th (**d**) IP injection of VEH, ABT263 (ABT) or PZ. The data presented are mean ± SEM. *a*, *b*, *c* *p* < 0.05 vs. Young, Aged + VEH and Aged + ABT, respectively. Expression of *Cdkn2a* (**e**)*, Cdkn1a* (**f**), *Il6 (***g**)*, Cxcl12 (***h**), *Mmp3* (**i**), and *Tnfsf11* (**j**) mRNA in the spleen, and expression of *Cdkn2a* mRNA in the liver (**k**), lung (**l**), kidney (**m**), and fat (**n**) of Young and naturally aged mice treated with VEH, ABT or PZ measured by quantitative PCR (qPCR) as illustrated in (**b**). The data presented are mean ± SEM. *a*, *b*, *c* *p* < 0.05 vs. Young, Aged + VEH and Aged + ABT, respectively. Data in **c**–**n** were analyzed by one-way ANOVA with Tukey’s post-hoc tests, or Kruskal–Wallis one-way ANOVA with Dunn’s post-hoc test, with *n* = 8, 8, 6, and 7 mice per group for Young, VEH-, ABT263-, and PZ-treated naturally aged mice, respectively. **n** *n* = 7, 7, 6, and 7 mice per group for Young, VEH-, ABT263-, and PZ-treated naturally aged mice, respectively. Source data are provided as a Source Data file. The exact *P* values are provided in the Source Data file.

To confirm that PZ can selectively kill SCs by functioning as a PROTAC to induce Bcl-xl degradation in a CRBN- and proteasome-dependent manner, we examined the effects of ABT263, pomalidomide (a CRBN ligand) or their combination on Bcl-xl levels in WI38 NCs and IR-SCs. None of these treatments affected Bcl-xl levels, suggesting that the effect of PZ on Bcl-xl is likely mediated through its PROTAC activity rather than the simple combination of ABT263 and pomalidomide (Fig. 2a). This suggestion is supported by the findings that: (1) pre-incubation of the cells with excess ABT263 or pomalidomide inhibited PZ-induced Bcl-xl degradation (Fig. 2b, c); (2) inhibition of proteasome activity with MG132 abolished the degradation of Bcl-xl induced by PZ (Fig. 2d); (3) PZ had no effect on the levels of Bcl-xl in CRBN knockout cells (Fig. 2e); and (4) Bcl-xl-NP, a PZ analog with an extra methyl group on the pomalidomide moiety that abrogates binding to CRBN (Supplementary Fig. 5), did not induce Bcl-xl degradation (Fig. 2f). In addition, the senolytic activity of PZ depended on its PROTAC activity because pomalidomide alone was not cytotoxic to WI38 NCs (Fig. 2g, left panel) or IR-SCs (Fig. 2g, right panel), nor did it have any additive or synergistic effect on WI38 IR-SC viability when combined with ABT263 (Fig. 2g, right panel). By contrast, the cytotoxicity of PZ against IR-SCs was reduced if CRBN was blocked by treating cells with a high concentration of pomalidomide prior to addition of PZ (Fig. 2h, right panel) and PZ was unable to reduce cell viability in CRBN knockout IR-SCs (Fig. 2i). Furthermore, Bcl-xl-NP was significantly less toxic to IR-SCs than PZ (Fig. 2j). Collectively, these data confirm that PZ acts as a PROTAC that depends on the CRBN E3 ligase and proteasome to degrade Bcl-xl and selectively induce IR-SC apoptosis.

### PZ clears SCs in aged mice without severe thrombocytopenia

Although PZ has a high molecular weight (MW = 1484.13 compared with 974.61 for ABT263) and low aqueous solubility, it is highly stable in plasma and in mouse and human liver microsomal stability assays (Supplementary Fig. 6a). Moreover, PZ has shown favorable pharmacokinetic (PK) properties and bioavailability by intraperitoneal injection (IP) or intravenous (IV) injection, but not by oral administration (PO) (Supplementary Fig. 6b, c). In addition, PZ was less toxic to platelets in mice than ABT263 after a single IP injection (Fig. 3a and Supplementary Fig. 6d) because mouse platelets also express low levels of CRBN and are insensitive to PZ-induced Bcl-xl degradation similar to human platelets (Supplementary Fig. 6e, f). Therefore, we tested whether IP injection of PZ can effectively clear SCs without causing severe thrombocytopenia in naturally aged mice compared with ABT263 at an equivalent effective dose (i.e. 41 μmol/kg). We used a less intensive dosing schedule (i.e., 41 μmol/kg, once every 3 days or q3d) for this experiment because PZ functioning as a PROTAC has a longer-acting activity than its corresponding inhibitor^39^ (Fig. 3b). One day after the first dose of ABT263 (41 μmol/kg or 40 mg/kg), severe thrombocytopenia occurred as expected, while injection of an equivalent dose (41 μmol/kg or 61 mg/kg) of PZ had less effect on blood platelet counts (Fig. 3c). Moreover, the severe thrombocytopenia induced by ABT263 was also observed 1 day after they received the 7th injection of ABT263, whereas the mice that received seven injections of PZ had just a mild reduction in blood platelet counts compared with vehicle-treated mice (Fig. 3d and Supplementary Table 1). Similar findings were also observed in total body irradiated (TBI) mice (Supplementary Fig. 7a–c). These findings demonstrate that PZ is less toxic to platelets than ABT263 in mice.

Next, we examined the ability of PZ to clear SCs in naturally aged mice in comparison with ABT263. We found that IP injections of PZ significantly decreased splenic expression of several SC biomarkers^40,41^, including *Cdkn2a* (*p16*), *Cdkn1a* (*p21*), and the SASP factors *Il6*, *Cxcl12*, *Mmp3,* and *Tnfsf11* (*Rankl*) mRNA (Fig. 3e–j), indicating that PZ can effectively clear SCs in naturally aged mice. ABT263 treatment was effective as PZ in reducing the expression of *Cdkn2a*, *Cdkn1a*, *Cxcl12*, and *Tnfsf11* mRNA but had no significant effect on the expression of *Il6* and *Mmp3* mRNA in the spleen (Fig. 3e–j). Moreover, PZ reduced the expression of *Cdkn2a* mRNA in the liver, lung, kidney, and fat in naturally aged mice, whereas ABT263 was less effective than PZ in reducing *Cdkn2a* mRNA expression in these organs (Fig. 3k–n). These results suggest that PZ may be slightly more effective than ABT263 in clearing SCs in mice. This suggestion is supported by the finding that under the same dosing regimen (41 μmole/kg, q3d) PZ was also slightly more effective than ABT263 in clearing SCs in the lungs of TBI mice (Supplementary Fig. 7d–g). However, at a more intensive dosing schedule, which consisted of two cycles of 7 daily IP injections of the same dose of either agent, ABT was equally effective to PZ in clearing SCs in multiple organs in naturally aged mice as shown in our previous studies (Supplementary Fig. 8)^16^. These results are in agreement with the unique mechanism of action of PROTACs, that usually have a longer-lasting effect and require less systemic drug exposure to be effective than their corresponding “payloads”. This is because PROTACs act catalytically to induce protein degradation in a sub-stoichiometric manner and their effect is not limited by equilibrium occupancy^32,39^.

### Clearance of SCs by PZ improves osteoprogenitor function

**Fig. 4:**
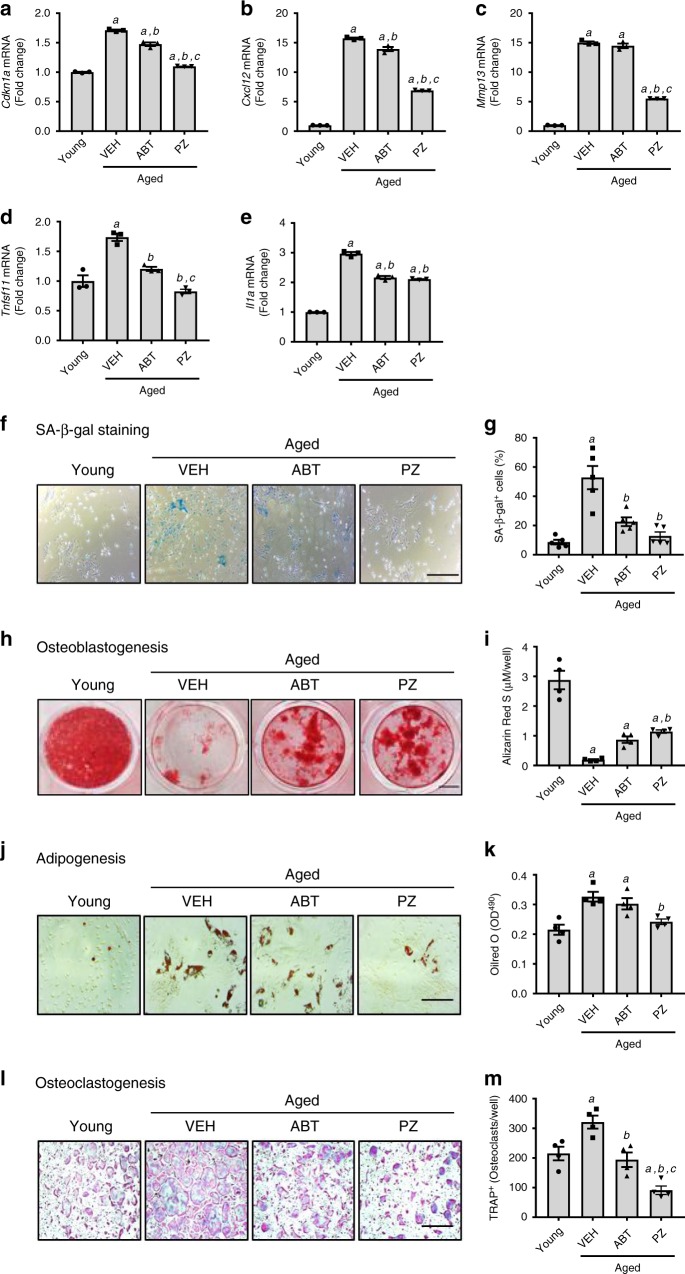
**PZ clears senescent bone marrow (BM) stromal cells to improve osteoprogenitor functions**. **a–f** Expression of *Cdkn1a* (**a**), *Cxcl12* (**b**), *Mmp13* (**c**), *Tnfsf11* (**d**) and *Il1α* (**e**) mRNA in BM stromal cells from young (Young) or naturally aged mice treated with vehicle (VEH), ABT263 (ABT) or PZ as illustrated in Fig. 3b was measured by quantitative PCR (qPCR). **f**, **g**. Analysis of senescent cells (SCs) in BM stromal cells from young (Young) and naturally aged mice treated with VEH, ABT, or PZ by senescence-associated β-galacotosidase (SA-β-gal) staining. Representative staining images (left) and percentage of SA-β-gal positive cells (right) are presented. **h**, **i** Analysis of osteoblastogenesis of BM stromal cells from young (Young) and naturally aged mice treated with VEH, ABT or PZ by Alizarin Red staining. Representative staining images (left) and quantification of Alizarin Red staining in the cells (right) are presented. **j**, **k**. Analysis of adipogenesis of BM stromal cells from young (Young) or naturally aged mice treated with VEH, ABT or PZ by Oil Red O staining. Representative staining images (left) and quantification of Oil Red O staining in the cells (right) are presented. **l**, **m**. Analysis of osteoclastogenesis of BM myeloid cells from young (Young) or naturally aged mice treated with VEH, ABT, or PZ by tartrate-resistant acid phosphatase (TRAP) staining. Representative images (left) and quantification of TRAP-positive multinucleated osteoclasts (right) are presented. The data are presented as mean ± SEM (**a**–**e**, *n* = 3 independent assays; **g**, n = 5 independent assays; **i**, **k**, and **m**, *n* = 4 independent assays; each assay using a sample pooled from 2 to 3 mice from the same experimental group). *a*, *b*, *c* *p* < 0.05 vs. Young, Aged + VEH, and Aged + ABT, respectively, determined by one-way ANOVA with Tukey’s post-hoc tests. Scale bars for **f**, **h**, **j**, and **l** are 50 µm, 1 cm, 50 µm, and 500 µm, respectively. Source data are provided as a Source Data file. The exact *P* values are provided in the Source Data file.

Recent studies by us and others have shown that SCs play an important role in skeletal aging in part by decreasing osteoprogenitor cell number and the generation of osteoblasts that produce the bone matrix; stimulate osteoclastogenesis to resorb the bone^42,43^. Senescence of bone marrow (BM) stromal cells also contributes to the increase in BM adipogenesis that occurs with age. Therefore, we examined whether PZ or ABT263 can counteract these effects of aging on bone cells by clearing SCs using the cells harvested from the mice shown in Fig. 3b. IP injection of PZ was slightly more effective than ABT263 in reducing the mRNA levels of *Cdkn1a* (Fig. 4a), *Cxcl12* (Fig. 4b), *Mmp13* (Fig. 4c), and *Tnfsf11* (Fig. 4d) in BM-derived osteoblast progenitor cell culture, but the expression of *Il1a* mRNA was reduced equally by PZ and ABT263 treatment (Fig. 4e). This result suggests that PZ might be slightly more effective than ABT263 in clearance of senescent BM osteoblast progenitors in naturally aged mice because these progenitors undergo senescence in a p21-dependent, but p16-independent, manner^42^. This suggestion is supported by the finding that PZ was also slightly more effective in reducing senescence-associated β-galactosidase (SA-β-gal) positive BM stromal cells than ABT263 (Fig. 4f, g). Because clearance of senescent BM osteoblast progenitors by PZ and ABT263 was associated with a significant reduction in the expression of the inflammatory cytokines IL1α and TNFSF11 that are known to play an important role in inhibiting bone formation and enhancing bone resorption^42,43^, we next examined the actions of PZ or ABT263 in regulating osteoblastogenesis, adipogenesis and osteoclastogenesis in naturally aged mice treated with vehicle, PZ or ABT263. We found that BM stromal cells from PZ-treated mice formed significantly higher amounts of mineralized matrix (Fig. 4h, i) and lower numbers of adipocytes than the cells from vehicle- or ABT263-treated mice (Fig. 4j, k). In addition, both PZ and ABT263 treatment decreased the osteoclastogenic potential of BM macrophages derived from BM myeloid cells (Fig. 4l, m). These findings indicate that both PZ and ABT263 can reduce the effects of aging on bone cells via clearance of SCs, however, PZ is slightly more effective than ABT263.

### PZ clears SCs and rejuvenates BM hematopoietic stem cells

**Fig. 5:**
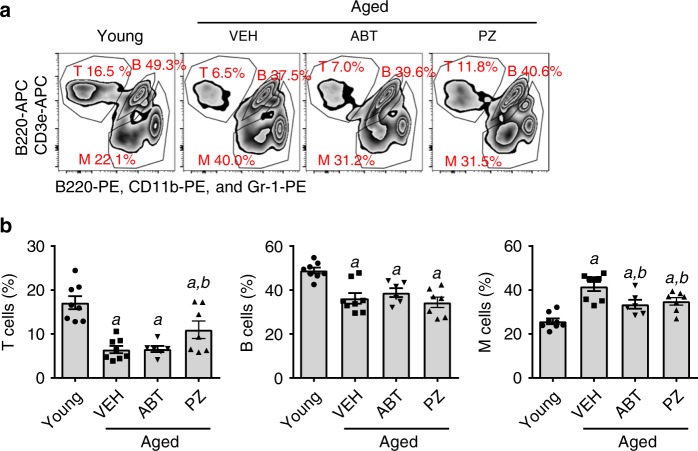
**PZ attenuates age-related myeloid skewing in naturally aged mice**. **a** Representative flow cytometric analyses of T cells (CD3e^+^), B cells (B220^+^) and myeloid cells (M cells; CD11b/Gr-1^+^) in blood from control untreated young mice (Young) or naturally aged mice treated with vehicle (VEH), ABT263 (ABT) or PZ as shown in Fig. 3b. **b** The percentages of blood T cells, B cells, and M cells in these mice are presented as mean ± SEM (*n* = 8, 8, 6, and 7 mice for Young mice and aged mice with VEH, ABT, and PZ treatment, respectively). *a*, *b* *p* < 0.05 vs. Young and Aged + VEH, respectively, determined by one-way ANOVA with Tukey’s or Dunnett’s post-hoc tests. Source data are provided as a Source Data file. The exact *P* values are provided in the Source Data file.

**Fig. 6:**
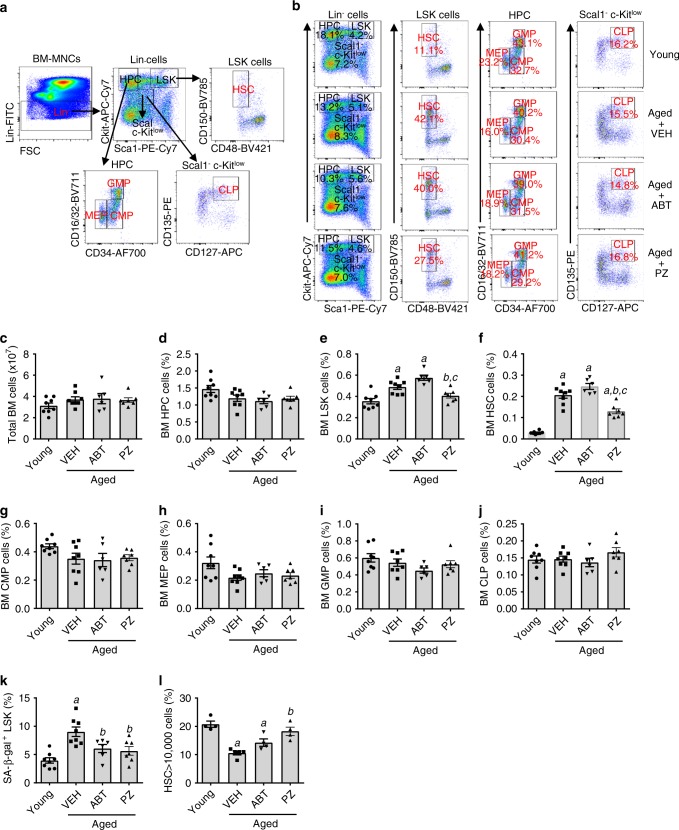
**PZ rejuvenates HSCs in naturally aged mice**. **a** A representative flow cytometric gating strategy of lineage negative (Lin^−^) cells, hematopoietic progenitor cells (HPC or Lin^−^sca1^–^c-kit^+^cells), LSK cells (Lin^–^sca1^+^c-kit^+^cells), and Scal1^–^c-Kit^low^ cells, HSC (Lin^–^sca1^+^c-kit^+^CD48^–^CD150^+^ cells), granulocyte–monocyte progenitors (GMP or Lin^–^sca1^–^c-kit^+^CD34^+^CD16/32^+^), megakaryocyte–erythrocyte progenitors (MEP or Lin^–^sca1^–^c-kit^+^CD34^–^CD16/32^–^), common myeloid progenitors (CMP or Lin^–^sca1^–^c-kit^+^CD34^+^CD16/32^–^), and common lymphoid progenitors (CLP or Lin^–^Sca1^–^c-Kit^low^ CD127^+^CD135^+^) in the bone marrow (BM) cells from control untreated young mice (Young) and naturally aged mice treated with vehicle (VEH), ABT263 (ABT) or PZ as shown in Fig. 3b. **b** Representative flow cytometric analyses of HPC, LSK cells, Sca1^–^c-Kit^low^ cells, HSC, CMP, MEP, CMP, and CLP in BM cells from the mice shown in Fig. 3b. Data are percentages of indicated cell counts in the cell populations labeled on the top of each panel. The number in each gate represents percentage of the gated cells in the cell population marked on the top of each panel. **c** Total BM cell counts in the mice shown in Fig. 3b. The percentages of BM HPC (**d**), LSK (**e**), HSC (**f**), CMP (**g**), MEP (**h**), GMP (**i**), and CLP (**j**) cells in total BM cells from the mice shown in Fig. 3b. **k**. Percentage of SA-β-gal positive senescent LSK cells in LSK cells from the mice shown in Fig. 3b. The data presented in **c**–**k** are mean ± SEM (*n* = 8, 8, 6, and 7 mice for the Young, VEH, ABT263, and PZ groups). *a*, *b*, *c* *p* < 0.05 vs. Young, Aged + VEH, and Aged + ABT, respectively. **l**. The percentage of individually sorted HSCs produced more than 10,000 cells in cultures. The data presented are mean ± SEM (*n* = 4 independent assays for Young, Aged + ABT, and Aged + PZ groups and n = 5 independent assays for Aged + VEH group). *a*, *b* *p* < 0.05 vs. Young, Aged + VEH, respectively. Data in **c** to **l** were analyzed by one-way ANOVA with Tukey’s post-hoc tests. Source data are provided as a Source Data file. The exact *P* values are provided in the Source Data file.

Cellular senescence also plays an important role in hematopoietic aging, including myeloid skewing (i.e. increase in myeloid cell differentiation and decrease in lymphoid differentiation) and expansion of phenotypic hematopoietic stem cells (HSCs) in part to compensate for the decrease in HSC function^27^. Our previous studies have shown that clearance of SCs with ABT263 rejuvenated HSCs to reduce age-associated myeloid skewing and HSC expansion in prematurely aged mice induced by TBI and naturally aged mice^16^. Therefore, we examined whether clearance of SCs by PZ could also rejuvenate HSCs in naturally aged mice from the experiment shown in Fig. 3b. PZ treatment attenuated age-associated myeloid skewing (Fig. 5a, b) and expansion of phenotypic HSCs (Fig. 6a–f), but had minimal effects on various hematopoietic progenitor cell populations that were not significantly affected by aging (Fig. 6g–j). PZ-induced significant reduction in SA-β-gal positive cells in HSC-enriched LSK (lineage negative and Sca1 and cKit positive) cells in naturally aged mice (Fig. 6k), which was associated with a significant increase in HSC clonogenicity (Fig. 6l) and a slightly improvement in HSC long-term engraftment (Supplementary Fig. 9). Thus, attenuation of hematopoietic aging might be in part attributable to the clearance of senescent HSCs. ABT263 treatment also attenuated age-associated myeloid skewing (Fig. 5), reduced SA-β-gal positive LSK cells (Fig. 6k), and increased HSC clonogenicity (Fig. 6l), but had no effect on age-associated HSC expansion (Fig. 6b, f). In addition, the increase in HSC clonogenic function was slightly less in ABT263-treated mice vs. PZ-treated mice. Again, these findings suggest that PZ might be more effective than ABT263 in rejuvenating aged HSCs under this less intensive treatment regimen because our previous study showed that ABT263 was more effective in rejuvenating aged HSCs under a more intensive treatment schedule^16^.

## Discussion

Here, we report an innovative strategy to reduce the on-target and dose-limiting tissue toxicity of a potent senolytic agent using PROTAC technology. Specifically, we converted a non-selective Bcl-2/xl dual inhibitor that is highly toxic to platelets into a Bcl-xl-specific PROTAC with lower toxicity to platelets. This was achieved by linking the Bcl-2/xl binding moiety of ABT263 to a CRBN ligand with an empirically optimized linker. The resulting Bcl-xl-specific PROTAC lead compound, PZ, selectively induced Bcl-xl degradation in NCs and SCs, but not platelets, because it targeted Bcl-xl to the CRBN E3 ligase that is poorly expressed in platelets but abundantly expressed in other cells^34,35^. In addition, the poor expression of CRBN-associated E1 (UBA1) and E2 (SFT) enzymes in platelets may also contribute to the inability of PZ to degrade Bcl-xl in platelets. Therefore, PZ can selectively induce apoptosis in SCs, but not platelets, which are dependent on Bcl-xl for survival while having minimal toxicity to NCs that are less sensitive to Bcl-xl inhibition than SCs^16–18,24–27^. More importantly, we found that PZ effectively cleared SCs in naturally aged mice without causing severe thrombocytopenia. The clearance of SCs resulted in a significant functional improvement of tissue stem and progenitor cells in multiple tissues in old mice.

Interestingly, after ABT263 is converted into PZ, the PROTAC becomes more specific to Bcl-xl and is incapable of inducing Bcl-2 and Bcl-w degradation. Similar phenomena have been reported previously. For example, the pan-bromodomain and extra-terminal (BET) inhibitor JQ1, which binds and inhibits the BET proteins BRD2, BRD3, and BRD4, could be converted into a selective BRD4 PROTAC^44^. In addition, Bondeson et al. showed that a promiscuous ligand for about 50 kinases could induce only a subset of its targets for degradation when converted into a PROTAC^45^. The lack of efficient degradation of Bcl-2 and Bcl-w by PZ may be in part attributed to the significant reduction of its binding affinity to these proteins. Alternatively, it could be due to consequences of under-optimization of the linker and linkage site, which could reduce its ability to form a stable target protein-PROTAC-E3 ligase ternary complex that is required for induction of target protein ubiquitination and degradation. In addition, Bcl-2 has fewer lysines than Bcl-xl, which may render Bcl-2 less sensitive than Bcl-xl to PZ-induced degradation^46^. The inability of PZ to efficiently induce Bcl-2 degradation may provide additional advantages for using PROTAC technology to reduce ABT263 on-target toxicity because Bcl-2 inhibition with a high dose or an intensive treatment regimen of ABT263 can lead to neutropenia and lymphocytopenia^25^. The lack of effect on Bcl-2 and Bcl-w expression by PZ also suggests that among various anti-apoptotic Bcl-2 family proteins, Bcl-xl is one of the most important survival factors for SCs. This suggestion is in agreement with previous findings that Bcl-xl-specific inhibitors such as A-1331852 and A-1155463 can selectively kill a variety of SCs^11^, whereas the Bcl-2 specific inhibitor ABT199 and Mcl-1 specific inhibitor A-1210477 had no such effect. However, inhibition of Bcl-2 and/or Bcl-w can potentiate the cytotoxicity of Bcl-xl inhibition against SCs as seen in previous studies with the dual Bcl-2/w and Bcl-xl inhibitors ABT737 and ABT263 (refs. ^16,17^).

Converting ABT263 to PZ slightly increases its senolytic activity to some types of SCs. This increase is likely due to PROTACs’ catalytical action to induce protein degradation in a sub-stoichiometric manner, and that their effect is not limited by equilibrium occupancy as are conventional inhibitors such as ABT263 (refs. ^29,30^). This unique property of PROTACs can potentially reduce off-target toxicities of ABT263 by lowering systemic concentration and duration of exposure, thus making Bcl-xl-Ps better senolytics than ABT263 or other Bcl-xl inhibitors. However, it is unlikely that PZ will be totally devoid of platelet toxicity, because it retains a high binding affinity to Bcl-xl (Ki = 1.90 nM for PZ vs. Ki = 1.53 nM for ABT263), and thus can still function as a Bcl-xl inhibitor that could potentially induce thrombocytopenia if the concentrations of PZ reach a high level in blood. This risk can be mitigated by additional optimization of the linker and linkage sites on the Bcl-xl and CRBN ligands to further improve the potency of PZ as a Bcl-xl PROTAC and by properly designing dosing regimen to avoid high blood concentrations that allow PZ to function as a Bcl-xl inhibitor. Targeting Bcl-xl to another E3 ligase, such as the von Hippe–Lindau (VHL), that is minimally expressed in platelets can also potentially improve the selectivity of Bcl-xl-Ps^34,35^. In addition, PZ is highly lipophilic and will require proper formulation and/or modifications to improve its drug-like properties as a senolytic agent to treat age-related diseases.

Our studies also confirm some of the previous observations that SCs from different cellular origins and tissues show variable sensitivities to different senolytic agents^2–5^. Although both PZ and ABT263 can be considered broad-spectrum senolytic agents, their effectiveness in clearing SCs in naturally aged mice appears to be tissue dependent. This observation is in agreement with the suggestion that different SCs can use different anti-apoptotic pathways (SCAPs) to resist apoptosis^2–5^. The effectiveness of SC clearance can be increased by increasing the dosage of PZ and ABT263 treatment, albeit at a cost of increasing drug toxicities. Therefore, it will be of a great interest to explore if PZ can be combined with other senolytic agents such as quercetin and/or dasatinib to synergistically kill SCs more effectively to treat age-related diseases without the risk of increasing drug toxicity^8,9^. This approach can potentially facilitate the translation of senolytic drug discovery research into clinic.

In summary, this study provides proof-of-principal evidence that it is possible to take advantage of tissue-specific expression of E3 ligases to achieve the induction of tissue- or disease-specific degradation of a target protein using the PROTAC technology to overcome on-target drug toxicity. This strategy may be useful to convert not only ABT263 but also other toxic repurposed senolytic agents into SC-selective PROTACs by employing an E3 ligase that is more abundantly expressed in SCs than normal tissues.

## Methods

### Chemical compounds and cytokines

Commercially available compounds and various cytokines used in the study are listed in Supplementary Table 2. The synthesis and analysis of PZ15227 (PZ) and Bcl-xl-NP are presented in Supplementary Note 1.

### Cell culture

Human WI38 fibroblasts (WI38, Cat. No. CCL-75), human IMR90 fibroblasts (IMR90, Cat. No. CCL-186), human renal epithelial cells (RECs, Cat. No. PCS-400-012) and human pre-adipocytes (PACs, Cat. No. PCS-210-010) were purchased from the American Type Culture Collection (ATCC, Manassas, VA, USA). WI38 and IMR90 cells were cultured in complete Dulbecco’s modified Eagle medium (DMEM, Cat. No. 12430054, Thermo Fisher Scientific, Waltham, MA, USA) supplemented with 10% heat-inactivated fetal bovine serum (FBS, Cat. No. S11150H, Atlanta Biologicals, Flowery Branch, GA, USA), 100 U/mL penicillin and 100 µg/mL streptomycin (Pen Strep, Cat. No. 15140122, Thermo Fisher Scientific) in a humidified incubator at 37 °C and 5% CO_2_. RECs were cultured in renal epithelial cell basal medium (Cat. No. PCS-400-030, ATCC) supplemented with complements from renal epithelial cell growth kit (Cat. No. PCS­400­040, ATCC). PACs were cultured in fibroblast basal medium (Cat. No. PCS-201-030, ATCC) supplemented with fibroblast growth kit-low serum (Cat. No. PCS­201­041, ATCC).

### SC induction

SCs were induced and validated as previously described^13,14,16^. Briefly, low-passage WI38 cells (<25 passages), IMR90 cells (<25 passages), RECs (<10 passages), and PACs (<4 passages) were used as NCs or for the induction of senescence. To induce SCs by ionizing radiation (IR-SCs), WI38, and IMR90 fibroblasts, RECs and PACs were treated as previously described^12–14,16^. To induce replicative senescence (RE-SCs), WI38 cells were subcultured until they stopped to divide and became permanently growth arrested or senescent after about 37 passages. To induce cellular senescence by ectopic expression of H-Ras (Ras-SCs), WI38 cells were infected with lentivirus containing pLenti CMV/TO RasV12 Puro (w119-1) (catalog no. 22262, Addgene, Cambridge, MA, USA). Two days after viral infection, transfected cells were selected by 2 μg/mL puromycin. Five days later, cells became senescent.

### Western blotting

After treatment with PZ or indicated compounds, cells were harvested in 1.5 mL microcentrifuge tubes and washed twice with ice-cold PBS. Protein was extracted using the RIPA buffer with EDTA and EGTA (Cat. No. BP-115DG, Boston BioProducts, Ashland, MA, USA) supplemented with 1% protease inhibitor cocktail (Cat. No. P8340, Sigma-Aldrich, St. Louis, MO, USA). Samples were put on ice for 30 min and then kept at −80 °C freezer overnight. After centrifugation at 15,000 × *g* at 4 °C for 20 min, supernatant was collected and the protein concentration was determined using the Pierce BCA protein Assay kit (Cat. No. 23225, Thermo Fisher Scientific). An equal amount of proteins (20–40 µg/lane) was loaded to a precast gel (Mini-PROTEAN TGX, Cat. No. 456-1094, Bio-Rad, Hercules, CA, USA) and transferred onto PVDF membranes (Invitrolon, Cat. No. LC2002, Life Technologies, Carlsbad, CA, USA) by electrophoresis. The membranes were blocked with 1X TBS-Tween (TBST, Cat. No. J77500, Affymetrix, Santan Clara, CA, USA) containing 5% non-fat dry milk (Cat. No. sc-2324, Santa Cruz Biotechnology, Dallas, TX, USA), and subsequently probed with primary antibodies at a predetermined optimal concentration overnight at 4 °C. After washing with TBST for 3 times (10 min each time), the membranes were incubated with the secondary horse radish peroxidase (HRP)-linked antibody for 1–2 h at room temperature. Following sufficient washing with TBST, the membranes were incubated with chemiluminescent HRP substrate (Cat. No. WBKLS0500, MilliporeSigma, Billerica, MA, USA). The blotting membranes were recorded using autoradiography (SRX-101, Konica, Shinjuku, Tokyo, Japan) or the ChemiDoc MP Imaging System (Bio-Rad, Hercules, CA, USA). Information on all antibodies used in western blot analyses is listed in Supplementary Table 3. The immunoblots were quantified using the ImageJ version 1.52a software.

### Cell viability assay

Number of viable cells was quantified using flow cytometry as previously described^13,14,16^. Specifically, NCs and SCs were incubated with vehicle (0.1% DMSO) or a test compound at indicated concentrations for indicated time points. Following dissociation with 0.25% Trypsin-EDTA (Cat. No. 25200056, Thermo Fisher Scientific) at 37 °C for 3–5 min, cells were harvested in 100 µL pre-cooled phosphate-buffered saline (PBS, Cat. No. 20012027, Thermo Fisher Scientific) containing 2% FBS and 100 ng/mL propidium iodide (PI, Cat. No. P4170, Sigma-Aldrich) and analyzed using flow cytometry (LSR II, BD Biosciences, San Jose, CA, USA). BD FACSDiva Software was used to collect the data. Percentage of viable cells were determined by counting the number of PI negative cells (viable cells) and then calculated as a ratio of control cells treated with vehicle^13,14,16^. Dose-response curves were generated for each compound, and the concentration for 50% of maximal effect (EC_50_ values) was calculated using GraphPad Prism 7 (GraphPad Software, La Jolla, CA, USA).

### Platelet isolation and viability assay

Human platelet-rich plasma (PRP) was purchased from Zenbio (Cat. No. SER-PRP-SDS, Research Triangle Park, NC, USA) within 3 days after harvest. PRP was transferred into a 50 mL tube containing 5 mL acid citrate buffer (Cat. No. sc-214744, Santa Cruz Biotechnology, Dallas, TX, USA). Mouse platelets were isolated from 10 to 16-week-old C57BL/6 J mice. Whole mouse blood was collected into a 50 mL polypropylene (PP) tube containing 5 mL citrate-dextrose solution (ACD) solution (Cat. No. sc-214744, Santa Cruz Biotechnology, Dallas, TX, USA) after euthanization. The anticoagulated blood was centrifuged at 250 × *g* for 20 min at room temperature without break. PRP was collected into a 15 mL polypropylene conical tube. To prevent clotting, prostaglandin E1 (PGE1, Cat. No. sc-201223A, Santa Cruz Biotechnology) and apyrase (Cat. No. A6237, Sigma-Aldrich) were added to final concentrations of 1 µM and 0.2 units/mL, respectively. After gently mixing the solution, platelets were pelleted by centrifugation at 1200 × *g* for 10 min without break. Pelleted platelets were gently washed without disrupting platelets in 2 mL HEPES Tyrode’s buffer (Cat. No. PY-921WB, Boston BioProducts, Ashland, MA, USA) containing 1 µM PGE1 and 0.2 units/mL apyrase. After washing, pellets were slowly suspended in 10 mL HEPES Tyrode’s buffer containing 1 µM PGE1, 0.2 units/mL apyrase and 10% FBS. Platelets number was then counted using the HEMAVET 950FS hematology analyzer (Drew Scientific, Miami Lakes, FL, USA). For viability assays, platelet number was adjusted to 2 × 10^8^/mL in HEPES Tyrode’s buffer containing 1 µM PGE1, 0.2 units/mL apyrase and 10% FBS. Each treatment was given in 2 mL platelet suspension in 15 mL polypropylene tubes. The tubes were placed on a rotating platform at room temperature and the viability of platelets was measured after treatment for indicated time points by using the MTS reagent (Cat. No. G1111, Promega, Madison, WI, USA). Through the whole process of the platelet viability assay, extra cautions such as room temperature storage/incubation, centrifuge without break, gently pipetting and mixing, and rotating platelets during incubation were taken to avoid platelet activation, aggregation, and spontaneous apoptosis. The data were analyzed by GraphPad Prism 7 software for the calculation of EC_50_ values.

### AlphaLISA assay

In order to evaluate the binding affinities of PZ and ABT263 towards Bcl-2, Bcl-xl, and Bcl-w, the AlphaScreen competitive binding assay was performed at room temperature and all reagents were diluted in a buffer containing 25 mM HEPES pH 7.5, 100 mM NaCl, 0.1% BSA, and 0.005% Tween-20. Purified recombinant His-tagged Bcl-xl, His-tagged Bcl-2 and His-tagged Bcl-w (Cat. No. SRP0187 for Bcl-xl, Cat. No. SRP0186 for Bcl-2 and Cat. No. B1059 for Bcl-w, Sigma-Aldrich) were incubated with increasing concentrations of compounds and a fixed concentration of biotin-tagged BAD (Cat No. SQ-ASPE-66592-1, AnaSpec, Fremont, CA) (for Bcl-xl) or BIM (Cat. No. SQ-ASPE-66592-2, AnaSpec) (for Bcl-2 and Bcl-w) peptides to a final volume of 40 µL in 96-well PCR plates. After 24 h incubation, 5 µL 6X His-Acceptor beads (final concentration 20 µg/mL) (Cat. No. AL128M, Perkin-Elmer, Waltham, MA, USA) were added to each well and incubated for 1 h. Thereafter, 5 µL streptavidin-donor beads were added (final concentration of 20 µg/mL) (Cat. No. 6760002, Perkin-Elmer) to each well and incubated for 30 min. At the end of the incubation period, 17 µL of each sample was transferred into adjacent wells of 384-well proxy plate (Cat. No. 6008280, Perkin-Elmer). The plate was scanned using Alpha program on a Biotek’s Synergy Neo2 multi-mode plate reader (Winooski, VT, USA). Gen5 version 3.04 software (BioTek, USA) was used for the AlphaLISA signal measurements. The inhibition constant (Ki) was calculated using non-linear regression, one site, competitive binding, fit Ki function on GraphPad Prism 7 software based on experimentally determined Kd for each protein/peptide pair.

### Apoptosis analysis

WI38 NCs and SCs were treated with vehicle or 10 µM Q-VD-OPh (QVD, Cat. No. S7311, Selleckchem, Houston, TX, USA) for 4 h prior to the addition of vehicle or PZ for 72 h. The suspended cells were first harvested from the culture and then pooled with the adherent cells detached by 0.25% Trypsin-EDTA at 37 °C for 3–5 min in 12 × 75 mm polystyrene round-bottom tubes (Cat. No. 352058, Falcon, Corning, NY, USA) containing complete DMEM medium. The cells were stained with Alexa Fluor 647-Annexin V (1: 50, Cat. No. 640912, BioLegend, San Diego, CA, USA) and PI (10 µg/mL, Cat. No. 421301, BioLegend, San Diego, CA, USA) at room temperature for 30 min. For analysis of platelet apoptosis, human platelets were washed with PBS containing 2% FBS and stained with Alexa Fluor 647 Annexin V (1:20) for 20 min at room temperature. The stained cells were analyzed on an Aurora flow cytometer and SpectroFlo Software was used to collect the data (Cytek Aurora, Fremont, CA, USA).

### CRBN knockout by CRISPR/Cas9 genomic editing

To knockout *CRBN*, the sgRNA targeting human *CRBN* was designed and cloned into a lentiCRISPR v2 vector (a gift from Feng Zhang; Addgene plasmid # 52961). Packaging 293 T cells were transfected with *CRBN* sgRNA or negative controls (non-targeting sgRNA-NC)^47^ and helper vectors (pMD2.G and psPAX2; Addgene plasmid #s 12259 and 12260) using Lipofectamine 2000 reagent (Cat. No. 11668019, Life Technologies). Medium containing lentiviral particles and 8 µg/mL polybrene (Cat. No. H9268, Sigma-Aldrich,) was used to infect WI38 cells. Infected cells were selected in medium containing 2 μg/mL puromycin. The target guide sequences are as follows: *sgCRBN*: forward (5′-CACCGAACCACCTGCCGCTCCTGCC-3′) and reverse (5′-AAACGGCAGGAGCGGCAGGTGGTTC-3′).

### Quantitative PCR (qPCR)

Total RNA was extracted using RNeasy Mini kit (Cat. No. 74106, QIAGEN, Gaithersburg, MD, USA). A total of 500–1000 ng mRNA was reversely transcribed into cDNA using the High Capacity cDNA Reverse Transcription kits (Cat. No. 4368813, Life Technologies) according to the manufacturer’s instructions. cDNA was then diluted with RNase free water by 5 times. Human *GAPDH*, mouse *Hprt* or mouse *Mrps2* was used as internal controls. Four μL diluted cDNA was mixed with 10 µL TaqMan Fast Advanced Master Mix (Cat. No. 4444965, Thermo Fisher Scientific) and 1 µL of Taqman assay probe (Supplementary Table 4). Samples were added 5 µL of H_2_O to have 20 µL of mixture and run qPCR according to the manufacturer’s instructions. All reactions were run in duplicate on an ABI StepOnePlus Real-Time PCR System (Applied Biosystems, Foster City, CA, USA). Target gene expression was calculated by normalizing to the housekeeping genes using the ∆Ct method^48^.

### Ubiquitination assay

Plasmid pSG5-Flag-Bcl-xl (pDL2009) was constructed by using Gibson Assembly Master Mix (NEB, Ipswich, MA, USA) and the following two primers sets:

primer set-1, forward (5′-GACTACAAGGACGACGATGACAAGGGATCTATGTCTCAGAGCAACCGGGAGCTGGTG-3′) and reverse (5′-GTTCTGCTTTAATAAGATCTGGATCTTCATTTCCGACTGAAGAGTGAGCCCAGCAGAAC-3′);

primer set-2, forward (5′-GTTCTGCTGGGCTCACTCTTCAGTCGGAAATGAAGATCCAGATCTTATTAAAGCAGAAC-3′) and reverse (5′-CACCAGCTCCCGGTTGCTCTGAGACATAGATCCCTTGTCATCGTCGTCCTTGTAGTC-3′).

pLX304-Bcl-xl (DNASU plasmid # HsCD00437924) and pSG5 (obtained from Invitrogen and Stratagene) were used as templates. Flag-Bcl-2 plasmid was a gift from Clark Distelhorst (Addgene plasmid #18003, Watertown, MA, USA). HA-Ub plasmid was a gift from Ted Dawson (Addgene, Plasmid #17608, Watertown, MA, USA). HEK293T cells (1.6 × 10^6^) were co-transfected with Flag-Bcl-xl and HA-Ub or Flag-Bcl-2 and HA-Ub for 40 h and then treated with DMSO, 1 μM ABT263 or 1 μM PZ15227 for 4 h. MG132 (10 μM) was added to prevent protein degradation in all cultures. Proteins were extracted by using immunoprecipitation lysis buffer (Cat. No. 87788, Thermo Fisher Scientific) and then subjected to immunoprecipitation using Anti-FLAG M2 Magnetic Beads (Cat. No. M8823, Sigma-Aldrich) according to the manufacturer’s protocol. Anti-FLAG M2 Magnetic Beads was washed with 1X TBS three times and then added to protein samples, and the mixture was incubated at 4 °C with rotation overnight. The magnetic beads were collected and then washed three times with 1X TBS. Immunoprecipitated samples were eluted with 2X SDS sample buffer and boiled 5 min at 95 °C. Proteins were analyzed by western blotting as described above.

### Naturally aged mice and treatments

Young (about 2 months old) male C57BL/6 J (or CD45.2) mice and B6.SJL-*Ptprc*^*a*^*Pep3*^*b*^/BoyJ (or CD45.1) mice (used as recipients for hematopoietic stem cell transplantation) were purchased from Jackson Lab (Bar Harbor, MA, USA). Breeding pairs of *p16-3MR* transgenic mice were kindly provided by Dr. Judith Campisi (Buck Institute for Research on Aging, Novato CA)^49^ and bred and maintained in the AAALAC-certified animal facility at the University of Arkansas for Medical Sciences (UAMS) and University of Florida (UF). They and their progeny received food and water *ad libitum*. Mice with tumors and/or leukemia were excluded from experiments and analyses. For the experiments presented in Figs. 3–6, 20-month-old male and female mice were randomly assigned to one of the treatment groups and received IP injections of vehicle (0.1 mL/mouse, q3d, 7 injections, *n* = 8 mice), ABT263 (41 μmole/kg or 40 mg/kg/q3d, 7 injections, *n* = 6 mice), or PZ (41 μmole/kg or 61 mg/kg/ q3d, 7 injections, *n* = 7 mice). ABT263 and PZ were formulated in 50% PHOSAL 50 PG, 45% MIGLYOL^®^ 810 N and 5% Polysorbate 80. A group of untreated young (2 months old, *n* = 8 mice) *p16-3MR* transgenic mice was included as controls. Various tissues were harvested for analyses after the mice were euthanized by CO_2_ suffocation and cervical dislocation 6 days after receiving the last injection. For the experiments presented in Supplementary Fig. 8, both male and female mice were randomly assigned to one of the treatment groups and received IP injections of vehicle (0.1 mL/mouse, 7 days per cycle for 2 cycles with a 2-week interval, *n* = 5 mice), ABT263 (41 μmole/kg or 40 mg/kg/day × 7 days per cycle for 2 cycles with a 2-week interval, *n* = 5 mice), or PZ (41 μmole/kg or 61 mg/kg/day × 7 days per cycle for 2 cycles with a 2-week interval, *n* = 5 mice). A group of untreated young (2 months old, *n* = 5 mice) *p16-3MR* transgenic mice was included as controls. Tissues were harvested for analyses after the mice were euthanized by CO_2_ suffocation and cervical dislocation 2 days after receiving the last injection.

### TBI mice and treatments

*p16***-***3MR* transgenic mice at 2–3 months of age were exposed to sham irradiation as controls or a sublethal dose (6.5 Gy) of TBI in a X-RAD 320 irradiator (Precision X-Ray, Branford, CT, USA) at a dose rate of 82 cGy/min. Fifteen weeks after TBI, four mice were treated with vehicle (50% PHOSAL 50 PG, 45% MIGLYOL^®^ 810 N and 5% Polysorbate 80), four mice with ABT263 (41 μmole/kg or 40 mg/kg, q3d/i.p), and five mice with PZ15227 (41 μmole/kg or 61 mg/kg, q3d/i.p.) for 3 weeks. A group of untreated (2–3 months old, n = 5 mice) *p16-3MR* transgenic mice was included as controls (CTL). Various tissues were harvested for analyses from above animal models after the mice were euthanized by CO_2_ suffocation and cervical dislocation 6 days after receiving the last injection.

### In vivo platelet toxicity assay

Female 5–6-week-old C57BL/6 mice were purchased from Jackson Lab (Bar Harbor, MA, USA) and treated with single IP injection of ABT263 (40 mg/kg) or PZ15227 (40 and 61 mg/kg). Approximately 50 μL of blood was collected from each mouse at 6 h, day 1, day 3, day 5, day 7, and day 10 via submandibular plexus route in EDTA tubes (Cat. No. 077051, RAM Scientific, Inc. Nashville, TN, USA) and platelets were enumerated using an automated hematology analyzer HEMAVET 950FS (Drew Scientific, Miami Lakes, FL, USA). For the mice used in Figs. 3–6 and Supplementary Fig. 7, mice were treated with indicated concentrations of ABT263 or PZ15227. Twenty-four hours after the first and last treatments, ~50 μL of blood was collected from each mouse into EDTA tubes through via submandibular plexus, and complete blood counts (CBCs) including platelets were immediately enumerated using HEMAVET 950FS (Drew Scientific, Miami Lakes, FL, USA).

All mice were housed in the Assessment and Accreditation of Laboratory Animal Care (AAALAC)-accredited animal facilities at UAMS or UF under pathogen-free conditions. All animal work was approved and done in accordance with the approvals from the Institutional Animal Care and Use Committees of UAMS and UF with the exception of the pharmacokinetic (PK) studies that were done by BioDuro (San Diego, CA, USA), a global contract research organization, through a contract. All animal studies were complied with the ethical regulations and humane endpoint criteria according to the NIH Guidelines for the Care and Use of Laboratory Animals.

### Analysis of blood T, B, and myeloid cells by flow cytometry

Blood T cells (CD3e^+^), B cells (B220^+^), and myeloid (M) cells (CD11b/Gr-1^+^) were stained with anti-CD3e, B220, Gr-1 and CD11b antibodies and analyzed on an Aurora flow cytometer (Cytek Aurora)^16,50^. Dead cells were excluded by gating out the cells stained positive with 7-Aminoactinomycin D (7-AAD, Cat. No. A1310, Thermo Fisher Scientific). The information for all these antibodies used in the staining was provided in Supplementary Table 5.

### Phenotypic analysis of BM cells by flow cytometry

The femora and tibiae were harvested from the mice immediately after they were euthanized. Bone marrow (BM) cells were flushed from the bones into PBS containing 2% FBS using a 21-gauge needle and syringe. BM mononuclear cells (BM-MNCs) were isolated and stained with the appropriate antibodies presented in Supplementary Table 5 to measure the frequencies of different cell types in BM, i.e., lineage negative (Lin^−^) cells, hematopoietic progenitor cells (HPC or Lin^−^sca1^−^c-kit^+^cells), LSK cells (Lin^−^sca1^+^c-kit^+^cells), and Scal1^−^c-Kit^low^ cells, HSC (Lin^−^sca1^+^c-kit^+^CD48^–^CD150^+^ cells), granulocyte–monocyte progenitors (GMP or Lin^–^sca1^–^c-kit^+^CD34^+^CD16/32^+^), megakaryocyte–erythrocyte progenitors (MEP or Lin^–^sca1^–^c-kit^+^CD34^–^CD16/32^–^), common myeloid progenitors (CMP or Lin^–^sca1^–^c-kit^+^CD34^+^CD16/32^–^) and common lymphoid progenitors (CLP or Lin^–^Sca1^–^c-Kit^low^ CD127^+^CD135^+^) using an Aurora flow cytometer (Cytek Aurora)^16,50^. FlowJo version 10 software was used to analyze the flow cytometry data.

### Isolation of BM HSCs

For the isolation of Lin^–^cells, BM-MNCs were incubated with biotin-conjugated rat antibodies specific for murine CD3e, CD11b, CD45R/B220, Ter-119, and Gr-1. The labeled mature lymphoid and myeloid cells were depleted by incubation with goat anti-rat IgG paramagnetic beads (Dynal Inc, Lake Success, NY, USA) at a bead: cell ratio of ~4:1. Cells binding the paramagnetic beads were removed with a magnetic field. The negatively isolated Lin^–^ cells were washed with 2% FBS/PBS, resuspended in 2% FBS/PBS at 1 × 10^7^/mL. LSK, HSCs and long-term HSCs were stained and isolated according the methods described previouly^16,50^. Dead cells were excluded by gating out 7-AAD (1:100, final concentration 10 μg/mL) positive cells. The sorted HSCs and LT-HSCs were cultured for single-cell colony-forming assay and competitive repopulation assay, respectively, as described below. The information for all these antibodies used in the staining was provided in Supplementary Table 5.

### Colony-forming cell assay in single HSC liquid culture

Single HSCs from individual mice were directly sorted into wells of round-bottom 96-well plates. The cells were cultured in freshly prepared RPMI 1640 culture medium supplemented with 10% FBS, 1% BSA, 1% penicillin and streptomycin, 2 mM L-Gln, 5 × 10^–5^ M β-mercaptoethanol, 10 ng/mL of recombinant mouse stem cell factor (mSCF), thrombopoietin (mTPO), and interleukin-3 (mIL-3). After 14 days of culture, the numbers of cells produced by each HSC were counted and the cells that produced more than 10,000 cells were scored as colony-forming cells. The information for mSCF, mTPO, and mIL-3 was provided in Supplementary Table 2.

### Competitive repopulation assay (CRA)

Fifty freshly sorted LT-HSCs from mice treated with vehicle or PZ were mixed with 3 × 10^5^ competitive BM cells pooled from three young CD45.1 mice and then transplanted into lethally irradiated (9.5 Gy TBI) CD45.1 recipients via retro-orbital injection of the venous sinus. Donor cell engraftment in the recipients was analyzed at 4 months after transplantation^16^.

### Senescence-associated-β-galactosidase (SA-β-gal) staining

BM LSK (Lin^−^sca1^+^c-kit^+^) cells were isolated according to the above protocol. SA-β-gal staining in LSK cells was performed by flow cytometry using an ImaGene Green C12FDG lacZ gene expression kit from Molecular Probes (Cat. No. I2904, Thermo Fisher Scientific) according to the manufacturer’s instructions and modified as previously described^16,50,51^.

### Osteoblast and adipocyte differentiation assays

Total BM cells were obtained by flushing the tibiae and femurs and pooled from more than 2–3 mice from each group. To analyze osteoblast progenitor cell activity, BM stromal cells were cultured with 20% FBS, 1% penicillin and streptomycin and 50 µg/mL of ascorbic acid (Cat. No. A4403, Sigma-Aldrich) in 10-cm culture dishes for 7 days. Half of the medium was replaced every 3 days. BM stromal cells were then re-plated in 12-well plates with 10% FBS, 1% penicillin and streptomycin, 50 µg/mL of ascorbic acid and 10 mM glycerophosphate (Cat. No. G9422, Sigma-Aldrich) for 5 days to perform qPCR assays or 21 days to perform Alizarin Red S staining. Mineralized matrix was stained with 40 mM alizarin red solution, following the manufacturer’s instructions (Cat. No. 40-1009, Sigma-Aldrich).

To analyze adipogenic potential, BM stromal cells were cultured to 80% confluence, and the media supplemented with rosiglitazone (5 nM/mL) or with 3.3% BSA in PBS as vehicle control. Medium was changed every 3–4 days. After 10 days, cells were fixed in 10% formalin in PBS, rinsed, and stained for 30 min with 0.15% Oil Red O (Cat. No. O0625, Sigma-Aldrich) in a 55:45 mix of isopropanol and water. Cells were counterstained with 0.5% methyl green (Cat. No. M295-25, Fisher Scientific, Pittsburgh, PA, USA) in 0.1 M sodium acetate, pH 4. Oil Red O staining was quantified after extraction of the dye with 1 mL isopropanol and absorbance determination at 490 nm. For all assays, cells were plated in triplicate.

### Osteoclast differentiation assay

BM cells were harvested as described above. After the red blood cells were removed with ACK buffer (0.01 mM EDTA, 0.011 M KHCO_3_ and 0.155 M NH_4_Cl, pH 7.3), we suspended the cells in α-MEM complete media containing 10% FBS, 1% penicillin and streptomycin and incubated the cells for 24 h in the presence of 10 ng/mL of M-CSF. Non-adherent BM myeloid were re-plated in Petri dishes with 30 ng/mL of M-CSF for 4 days to generate BM macrophages (BMMs), which were used as osteoclast precursors. To generate mature osteoclasts, BMMs were cultured with 30 ng/mL of M-CSF and 30 ng/mL of RANKL for 4–5 days. The medium was replaced every 3 days. Osteoclasts were fixed with 10% neutral buffered formalin for 15 min and stained for tartrate-resistant acid phosphatase (TRAP), using the Leukocyte Acid Phosphatase Assay Kit (Cat. No. 386 A, Sigma-Aldrich), following the manufacturer’s instructions. An osteoclast was defined as a multinuclear (more than 3 nuclei) TRAP-positive cell. For all assays, cells were plated in triplicate.

### SA-β-gal staining in BM stromal cells

SA-β-gal activity was detected using the Senescence β-Galactosidase Staining Kit (Cat. No. 9860, Cell Signaling Technology, Danvers, MA, USA) according to the manufacturer’s instructions.

### Statistical analysis

All statistical analyses were performed with Graphpad Prism 7. All data are presented as means ± SEM (or SD if the data are from replications of a representative experiment but not from independent assays). Comparisons were made by two-tailed Student’s *t*-test when comparing two experimental groups. For a comparison between more than two groups, one-way ANOVA with Tukey’s or Dunnett’s post-hoc tests was used. For one-way ANOVA analysis that failed the normality test, Kruskal–Wallis one-way ANOVA with Dunn’s post-hoc test was performed. For Fig. 2h–j, two-way ANOVA with Tukey’s post-hoc tests was used. All western blots showed in the figures were repeated in at least two independent experiments. *P* < 0.05 was considered to be significant. The exact *P* values were provided in the Source Data file.

### Reporting summary

Further information on research design is available in the Nature Research Reporting Summary linked to this article.

## Supplementary information

Reporting Summary

Supplementary Information

## Data Availability

The source data underlying Figs. 1–6 are provided as a Source Data file. The data sets generated and/or analyzed during the current study are available from the corresponding authors upon reasonable request.

## Source Data

Source Data
