# Supplementary material for: Using proteolysis-targeting chimera technology to reduce navitoclax platelet toxicity and improve its senolytic activity
Source: Nat Commun. 2020 Apr 24;11:1996. doi: 10.1038/s41467-020-15838-0 (PMC7181703; doi:10.1038/s41467-020-15838-0)
Supplement: Supplementary file 1 — Reporting Summary [file 41467_2020_15838_MOESM1_ESM.pdf]

nature research

Corresponding author(s): Daohong Zhou, Guangrong Zheng

Last updated by author(s): Oct 17, 2019

Reporting Summary

Nature Research wishes to improve the reproducibility of the work that we publish. This form provides structure for consistency and transparency in reporting. For further information on Nature Research policies, see Authors & Referees and the Editorial Policy Checklist .

Statistics

For all statistical analyses, confirm that the following items are present in the figure legend, table legend, main text, or Methods section.

n/a	Confirmed	
☐	☒	The exact sample size (*n*) for each experimental group/condition, given as a discrete number and unit of measurement
☐	☒	A statement on whether measurements were taken from distinct samples or whether the same sample was measured repeatedly
☐	☒	The statistical test(s) used AND whether they are one- or two-sided<br>*Only common tests should be described solely by name; describe more complex techniques in the Methods section.*
☐	☒	A description of all covariates tested
☐	☒	A description of any assumptions or corrections, such as tests of normality and adjustment for multiple comparisons
☐	☒	A full description of the statistical parameters including central tendency (e.g. means) or other basic estimates (e.g. regression coefficient) AND variation (e.g. standard deviation) or associated estimates of uncertainty (e.g. confidence intervals)
☐	☒	For null hypothesis testing, the test statistic (e.g. *F*, *t*, *r*) with confidence intervals, effect sizes, degrees of freedom and *P* value noted<br>*Give P values as exact values whenever suitable.*
☒	☐	For Bayesian analysis, information on the choice of priors and Markov chain Monte Carlo settings
☒	☐	For hierarchical and complex designs, identification of the appropriate level for tests and full reporting of outcomes
☒	☐	Estimates of effect sizes (e.g. Cohen's *d*, Pearson's *r*), indicating how they were calculated

Our web collection on statistics for biologists contains articles on many of the points above.

Software and code

Policy information about availability of computer code

Data collection	The immunoblots were scanned using autoradiography (Konica, Japan) or were recorded using the ChemiDoc MP Imaging System (Bio-Rad, USA). Flow cytometry data were collected using BD flow cytometry (LSR II, BD Biosciences), Aria II cell sorter (BD Biosciences, USA) or Aurora flow cytometer (Cytek Aurora, USA). BD FACSDiva™ Software and SpectroFlo® Software were used to collect data on BD flow cytometers and Aurora flow cytometer, respectively. Gen5 version 3.04 software (BioTek, USA) was used for absorbance or luminescence measurements in 96-well plates on Synergy Neo2 multi-mode plate reader. Blood cells (including platelet) were counted using the HEMAVET 950FS hematology analyzer (Drew Scientific, USA). Quantitative PCR were run on an ABI StepOnePlus Real-Time PCR System (Applied Biosystems, USA).
Data analysis	The immunoblots were quantified using the ImageJ version 1.52a software (NIH). GraphPad Prism version 7 (GraphPad Software, USA) was used for the preparation of all the graphs, determination of half maximal effective concentration (EC50) values, Inhibition constant (Ki) and the statistical analyses. FlowJo version 10 software was used to analyze the flow cytometry data.

For manuscripts utilizing custom algorithms or software that are central to the research but not yet described in published literature, software must be made available to editors/reviewers. We strongly encourage code deposition in a community repository (e.g. GitHub). See the Nature Research guidelines for submitting code & software for further information.

Data

Policy information about availability of data

All manuscripts must include a data availability statement. This statement should provide the following information, where applicable:

- Accession codes, unique identifiers, or web links for publicly available datasets
- A list of figures that have associated raw data
- A description of any restrictions on data availability

The source data underlying Figs. 1-6 are provided as a Source Data file. The data sets generated and/or analyzed during the current study are available from the corresponding author upon reasonable request.

October 2018

Field-specific reporting

Please select the one below that is the best fit for your research. If you are not sure, read the appropriate sections before making your selection.

☒ Life sciences ☐ Behavioural & social sciences ☐ Ecological, evolutionary & environmental sciences

For a reference copy of the document with all sections, see nature.com/documents/nr-reporting-summary-flat.pdf

Life sciences study design

All studies must disclose on these points even when the disclosure is negative.

Sample size	Sample sizes were designed based on prior assay experience (Nat Med. 2016 Jan;22(1):78-83), similar work on related projects, and pilot data.
Data exclusions	In Fig. 3n, a sample in Young group and a sample in VEH group were excluded due to failure of mRNA extraction. An outlier in PZ group was identified by the Graphpad Prism software and excluded from the analyses.
Replication	Adequate measures were taken to verify the reproducibility of findings. An effect was confirmed across multiple cell lines, senescent types with independent experiments and using several biological and technical replicates. The compounds were tested at multiple concentrations in vitro and the in vivo results were confirmed in different model systems such as in naturally aged mice and total body irradiated (TBI) mice. Some results were confirmed by different investigators and in different laboratory conditions. All attempts at replication were successful.
Randomization	Animals were randomized in different groups on the basis of body weight in a way that each group had nearly equal average body weight at the start of treatment.
Blinding	Investigators were not blinded during randomizing the mice in different groups, because the body weight of the aged mice are not distributed evenly even the mice had the same age. The animals were segregated into separate labeled cages according to the body weight to ensure that each group had animals with similar body weight. For in vitro experiments, investigators were not blinded to the group allocation during the data collection and analysis, because the blinding was not relevant to the study.

Reporting for specific materials, systems and methods

We require information from authors about some types of materials, experimental systems and methods used in many studies. Here, indicate whether each material, system or method listed is relevant to your study. If you are not sure if a list item applies to your research, read the appropriate section before selecting a response.

Materials & experimental systems

n/a	Involved in the study	
☐	☒	Antibodies
☐	☒	Eukaryotic cell lines
☒	☐	Palaeontology
☐	☒	Animals and other organisms
☒	☐	Human research participants
☒	☐	Clinical data

Methods

n/a	Involved in the study	
☒	☐	ChIP-seq
☐	☒	Flow cytometry
☒	☐	MRI-based neuroimaging

Antibodies

Antibodies used

Antibodies for Western blot analyses:
Antibody Clone Antibody isotype Catalog # Concentration
Bcl-xl _ Rabbit IgG Polyclonal 2762S 1:1000
Mcl-1 D35A5 Rabbit IgG Monoclonal 5453S 1:1000
Bcl-2 50E3 Rabbit IgG Monoclonal 2870S 1:1000
β-actin 13E5 Rabbit IgG Monoclonal 4970S 1:1000
CRBN _ Rabbit IgG Polyclonal HPA045910 1:1000
UBA1 _ Rabbit IgG Polyclonal 4890S 1:1000
SFT _ Rabbit IgG Polyclonal ab176561 1:5000
Bcl-w 31H4 Rabbit IgG Polyclonal 2724S 1:1000
Glutamine synthetase E-4 Mouse IgG Monoclonal SC-74430 1:1000
CK1α _ Rabbit IgG Polyclonal 2655S 1:1000
IKZF1 _ Rabbit IgG Polyclonal 5443S 1:1000
IKZF3 D1C1E Rabbit IgG Monoclonal 15103S 1:1000
Flag tag _ Rabbit IgG Polyclonal 2044S 1:1000
HA tag C29F4 Rabbit IgG Monoclonal 14031S 1:1000

β-actin C4 Mouse IgG Monoclonal 8691001 1:10000
Secondary antibody _ Anti-rabbit IgG 7074S 1:3500
Secondary antibody _ Anti-mouse IgG 7076S 1:5000

Antibodies for flow cytometry and cell sorting:
Antibody Clone Antibody isotype Conjugate Catalog # Concentration
CD45R/B220 RA3-6B2 IgG2a purified 553084 1:200
CD3e 145-2C11 IgG1 purified 553238 1:200
CD11b M1/70 IgG2b purified 553308 1:200
Gr-1 RB6-8C5 IgG2b purified 553123 1:200
Ter-119 Ter-119 IgG2b purified 553671 1:200
CD45R/B220 RA3-6B2 IgG2a biotin 553086 1:200
CD3e 145-2C11 IgG1 biotin 553239 1:200
CD11b M1/70 IgG2b biotin 553309 1:200
Gr-1 RB6-8C5 IgG2b biotin 553125 1:200
Ter-119 Ter-119 IgG2b biotin 553672 1:200
CD45.1 A20 IgG2a FITC 553775 1:200
CD45.2 104 IgG2a Alexa Fluor® 700 560693 1:100
CD45R/B220 RA3-6B2 IgG2a APC 553092 1:200
CD45R/B2201RA3-6B2 IgG2a PE 553090 1:200
CD11b M1/70 IgG2a PE 553311 1:200
Gr-1 RB6-8C5 IgG2a PE 561083 1:200
CD34 RAM34 IgG2a Alexa Fluor® 700 560518 1:30
CD48 HM48-1 IgG1 BV421 562745 1:200
CD135(Flt3) A2F10 IgG2a PE 553842 1:200
CD127(IL7) A019D5 IgG1 APC 564175 1:200
Streptavidin - - FITC 554060 1:100
Sca-1 E13-161.7 IgG2a PE-Cy7 558162 1:200
c-kit 2B8 IgG2b APC-Cy7 560185 1:200
CD150 9D1 IgG2a BV785 115937 1:200
CD3e 145-2C11 IgG APC 553311 1:200
CD16/32 93 IgG2a BV711 101337 1:200

Validation

All the used antibodies are commercially available. The antibodies used in a specific species or application have been validated by manufacturers to be used in that species/application and this information is provided in their website and/or antibody datasheets.

Eukaryotic cell lines

Policy information about cell lines

Cell line source(s)

Human WI38 fibroblasts (WI38, Cat. No. CCL-75), human IMR90 fibroblasts (IMR90, Cat. No. CCL¬-186), human renal epithelial cells (RECs, Cat. No. PCS-400-012) and human pre-adipocytes (PACs, Cat. No. PCS-210-010) were purchased from the American Type Culture Collection (ATCC, Manassas, VA, USA).

Authentication

The cell lines have been validated by the suppliers. The morphology and growth of the used cell lines were verified with the supplier's data sheets, and their pharmacological responses were matched with the available literature.

Mycoplasma contamination

All cell lines tested negative for mycoplasma contamination when purchased from ATCC.

Commonly misidentified lines
(See ICLAC register)

No commonly misidentified cell lines were used.

Animals and other organisms

Policy information about studies involving animals; ARRIVE guidelines recommended for reporting animal research

Laboratory animals

Female 5-6 weeks old C57BL/6 mice were purchased from from Jackson Lab (Bar Harbor, MA, USA) and were used for the in vivo platelet toxicity assay. Young (about 2 months old) male C57BL/6J (or CD45.2) mice and B6.SJL-PtprcaPep3b/BoyJ (or CD45.1) mice (used as recipients for hematopoietic stem cell transplantation) were purchased from Jackson Lab (Bar Harbor, MA, USA). Breeding pairs of p16-3MR transgenic mice were kindly provided by Dr. Judith Campisi (Buck Institute for Research on Aging, Novato CA). All mice were housed in the Assessment and Accreditation of Laboratory Animal Care (AAALAC)-accredited animal facilities at Arkansas for Medical Sciences (UAMS) or University of Florida (UF) under pathogen-free conditions. They and their progeny received food and water ad libitum. Mice with tumors and/or leukemia were excluded from experiments and analyses. 20 months or older male and female mice were considered as naturally aged mice and were randomly assigned to one of the treatment groups.

Wild animals | The study did not involve wild animals.

Field-collected samples | The study did not involve samples collected from the field.

Ethics oversight | All animal work was approved and done in accordance with the Institutional Animal Care and Use Committees of University of Arkansas for Medical Sciences and University of Florida, except the pharmacokinetic (PK) studies that were done by BioDuro (San Diego, CA), a global contract research organization, through a contact. All animal studies were complied with the ethical regulations and humane endpoint according to the NIH Guidelines for the Care and Use of Laboratory Animals.

Note that full information on the approval of the study protocol must also be provided in the manuscript.

Flow Cytometry

Plots

Confirm that:

- [x] The axis labels state the marker and fluorochrome used (e.g. CD4-FITC).
- [x] The axis scales are clearly visible. Include numbers along axes only for bottom left plot of group (a 'group' is an analysis of identical markers).
- [x] All plots are contour plots with outliers or pseudocolor plots.
- [x] A numerical value for number of cells or percentage (with statistics) is provided.

Methodology

Sample preparation | For the cell counting in-vitro experiment, following dissociation with 0.25% Trypsin-EDTA at 37°C for 3-5 min, cells were harvested in 100 µL pre-cooled phosphate-buffered saline containing 2% FBS and 100 ng/mL propidium iodide (PI) and analyzed using flow cytometry. For apoptosis assay, cells were stained with Alexa Fluor 647-Annexin V and PI at room temperature for 30 min. For the in-vivo experiment, the femora, tibiae and blood were harvested from the mice immediately after they were euthanized. Bone marrow cells, myeloid and lymphoid cells were analyzed or sorted according to their special antigen markers, which were described in detail in the "Methods".

Instrument | LSR II (BD Biosciences), Aria II cell sorter (BD Biosciences), Aurora flow cytometer (Cytek Aurora)

Software | FlowJo, V10

Cell population abundance | For mouse samples, at least 50,000 events of live cells were collected. For apoptosis assay, at least 5000 cells were collected.

Gating strategy | FSC-A/SSC-A was used to distinguish the cell size. PI or 7-AAD negative was used to gate the live cells. FSC-A and FSC-H was used to gate the single cells, all single live cells were analyzed.

- [x] Tick this box to confirm that a figure exemplifying the gating strategy is provided in the Supplementary Information.
