# Supplementary material for: Using proteolysis-targeting chimera technology to reduce navitoclax platelet toxicity and improve its senolytic activity
Source: Nat Commun. 2020 Apr 24;11:1996. doi: 10.1038/s41467-020-15838-0 (PMC7181703; doi:10.1038/s41467-020-15838-0)
Supplement: Supplementary file 2 — Supplementary Information [file 41467_2020_15838_MOESM2_ESM.pdf]

Using proteolysis targeting chimera technology to reduce navitoclax platelet toxicity and improve its senolytic activity

He Y et al.

**Supplementary Item**	**Title**
Supplementary Figure 1	PZ15227 (PZ) is a Bcl-xl specific PROTAC
Supplementary Figure 2	PZ is less toxic to human platelets (PLTs) than ABT263 (ABT)
Supplementary Figure 3	PZ induced apoptosis selectively in WI38 IR-SCs but not in NCs in a caspase-dependent manner
Supplementary Figure 4	PZ is a potent and broad-spectrum senolytic agent
Supplementary Figure 5	Comparison of the structures of PZ and Bcl-xl-NP
Supplementary Figure 6	Preliminary drug metabolism and pharmacokinetics (DMPK) and platelet toxicity studies in mice
Supplementary Figure 7	PZ can effectively clear SCs in total body irradiated (TBI) mice without causing significant thrombocytopenia
Supplementary Figure 8	A more intense dosing regimen of ABT263 (ABT) treatment can equally effective clear SCs as PZ in naturally aged mice
Supplementary Figure 9	PZ rejuvenates the function of HSCs in naturally aged mice
Supplementary Table 1	Hematology profile in young and naturally aged mice treated by vehicle (VEH), ABT263 (ABT) or PZ15227 (PZ)
Supplementary Table 2	Suppliers for various compounds and cytokines
Supplementary Table 3	Antibodies for western blot analyses
Supplementary Table 4	TaqMan probes used for qRT-PCR
Supplementary Table 5	Antibodies for flow cytometry and cell sorting
Supplementary Note 1	Procedures for the Synthesis of PZ15227 (PZ) and Bcl-xl-NP

a

Binding affinity

Ki	Bcl-xl (nM)	Bcl-2 (nM)	Bcl-w (nM)
PZ	1.90 ± 0.15	3.52 ± 0.26	>1000
ABT	1.53 ± 0.07	1.03 ± 0.11	8.5

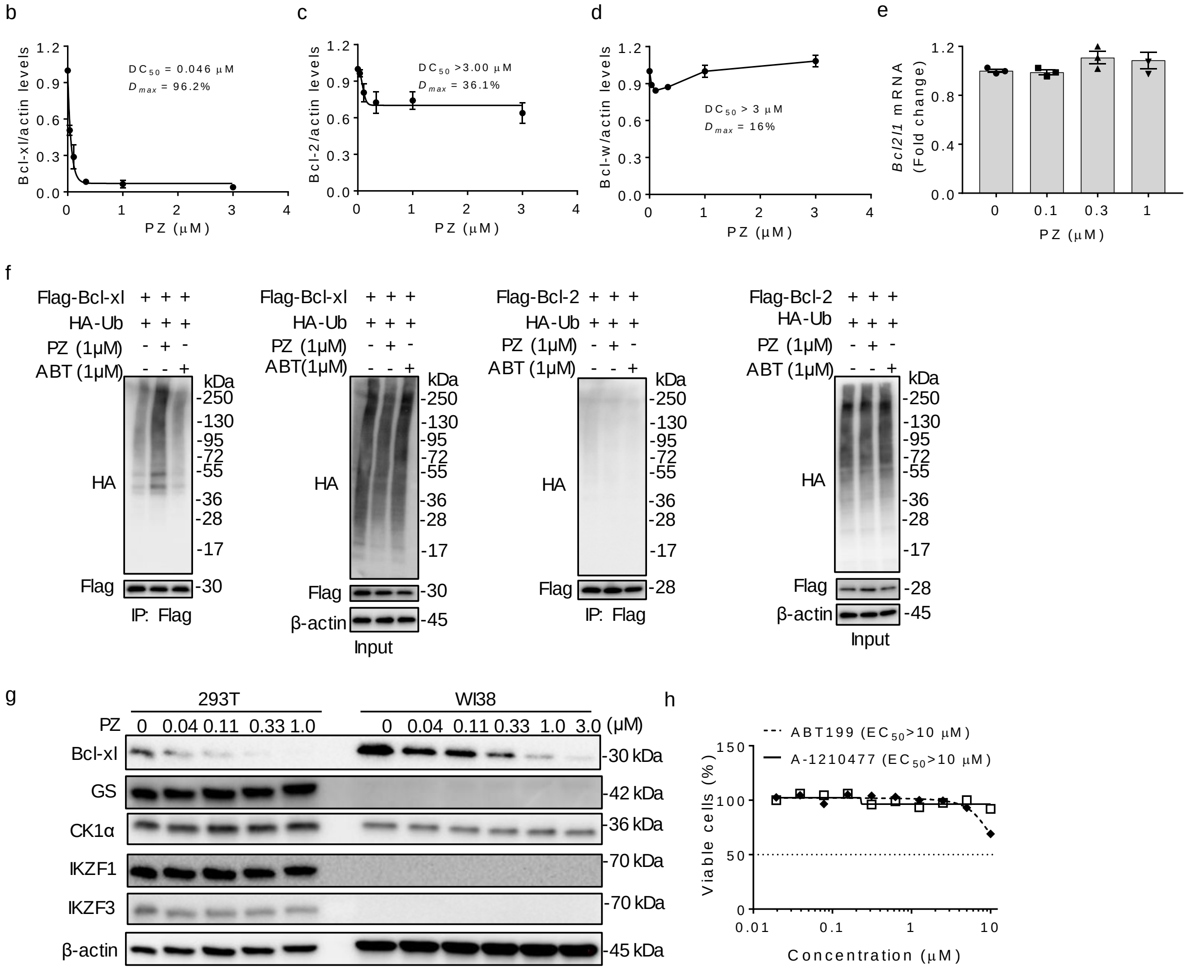

Supplementary Figure 1. PZ15227 (PZ) is a Bcl-xl specific PROTAC. a. The binding affinities of PZ and ABT263 (ABT) to Bcl-xl (n = 3 and 4 independent experiments for PZ and ABT, respectively), Bcl-2 (n = 4 and 6 independent experiments for PZ and ABT, respectively) and Bcl-w (n = 2 replicates in one experiment). The data are presented as mean ± SEM. **b-d.** The levels of Bcl-xl (**b**), Bcl-2 (**c**) and Bcl-w (**d**) in WI38 cells quantified by western blots show that PZ induced degradation of Bcl-xl but not Bcl-2 and Bcl-w in a dose-dependent manner. DC_{50}, the half-maximal degradation concentration; D_{max}, the maximum level of protein degradation. The data are presented as mean ± SEM (n = 4, 3 and 2 independent experiments for **b**, **c**, and **d**, respectively). **e.** PZ had no effect on the expression of *Bcl2l1* mRNA in WI38 cells. The levels of *Bcl2l1* mRNA

were measured by qPCR. The data are presented as mean ± SD from a representative assay (n = 3 replicates). **f.** PZ, but not ABT, selectively induced ubiquitination of Flag-Bcl-xl (panel 1 and 2), but neither PZ nor ABT had any effect on Flag-Bcl-2 ubiquitination (panel 3 and 4) in 293T cells. HA-Ub, HA ubiquitin. Similar results were got in at least two independent experiments. **g.** PZ did not reduce the expression of other known CRBN substrates in 293T or WI38 cells. GS, glutamine synthetase; CK1α, Casein kinase I alpha; IKZF1, IKAROS Family Zinc Finger 1; and IKZF3, IKAROS Family Zinc Finger 3. A representative immunoblot is presented. Similar results were got in at least two independent experiments. **h.** The percentages of viable cells in WI38 senescent cells (SCs) induced by irradiation (IR-SCs) after treatment with different concentrations of Bcl-2 specific inhibitor ABT199 or Mcl-1 specific inhibitor A-1210477 for 72 h. The data are presented as mean value (n = 2 independent experiments).

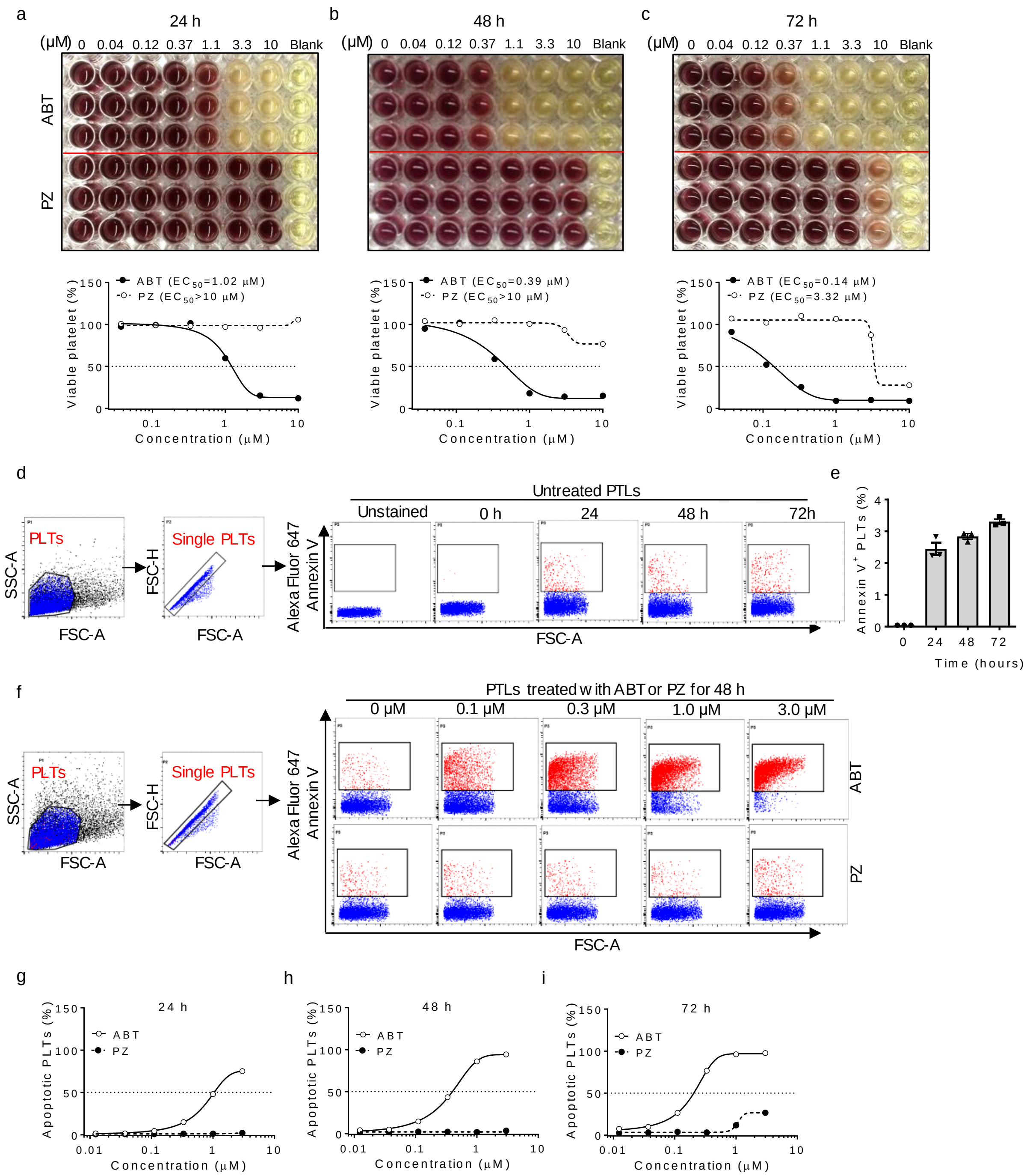

Supplementary Figure 2. PZ is less toxic to human platelets (PLTs) than ABT263 (ABT). a-c. ABT but not PZ dose-dependently reduced the viability of PLTs. Top panels are images of representative MTS assays to measure PLT viability after treatment with indicated concentrations of ABT or PZ for 24, 48 or 72 h. Bottom panels show percentage of viable PLTs quantified from the top panels. The data are presented as mean ± SD (n = 3 replicates from a representative

experiment). **d.** Representative flow cytometric analyses of spontaneous apoptosis in PLTs by Alexa Fluor 647-Annexin V staining after they were cultured at room temperature for indicated time points. **e.** Quantification of spontaneous PLT apoptosis from **d**. The data are presented as mean ± SD of percentage of apoptotic PLTs (n = 3 replicates from a representative experiment). **f.** Representative flow cytometric analyses of apoptosis in PLTs by Alexa Fluor 647-Annexin V staining after they were cultured with different concentrations of ABT or PZ for 48 h. **g.** Percentage of apoptotic PLTs after they were treated with different concentrations of ABT or PZ for 24 h, 48 h or 72 h. The data are presented as mean ± SD of percentages of apoptotic PLTs (n = 3 replicates from a representative experiment).

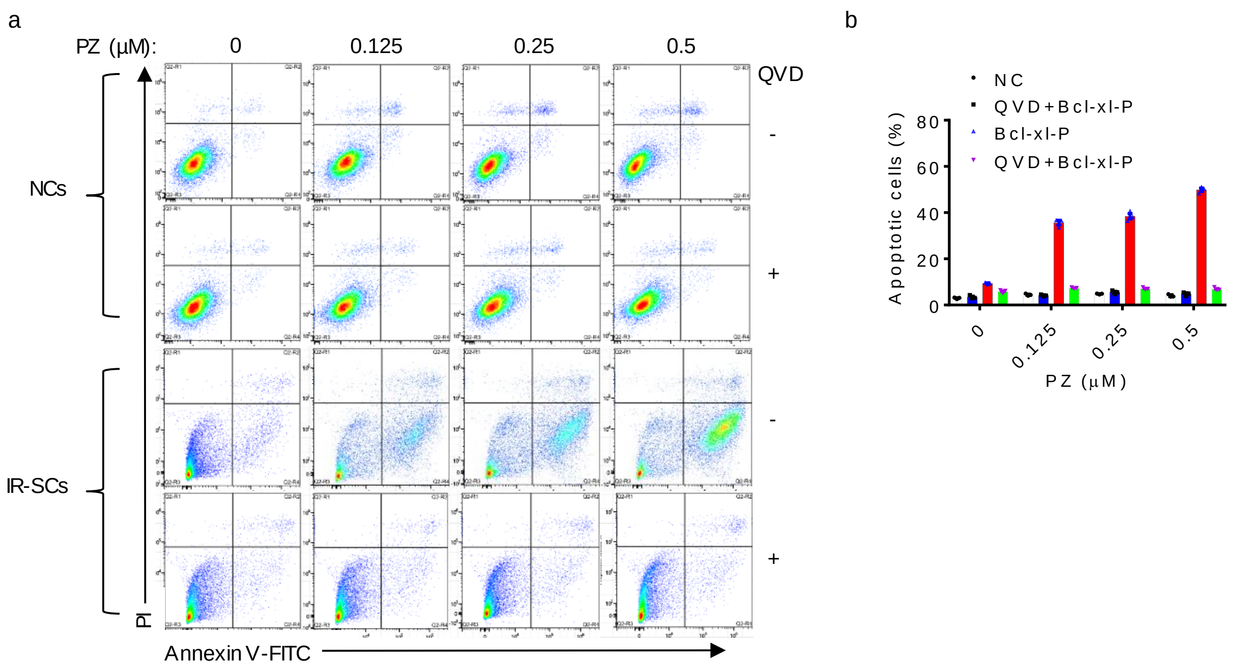

Supplementary Figure 3. PZ induced apoptosis selectively in WI38 IR-SCs but not in WI38 non-SCs (NCs) in a caspase-dependent manner. a. Representative flow cytometric assays for apoptosis. **b.** The percentage of apoptotic cells (Annexin V$^+$ and Annexin V$^+$PI$^+$ cells) shows that PZ dose-dependently induced apoptosis in IR-SCs but not in NCs, which could be abrogated by pretreatment with the pan-caspase inhibitor QVD (10 µM) for 4 h prior to PZ treatment. The data are presented as mean ± SD of percentage of apoptotic cells (n = 3 replicates from a representative experiment).

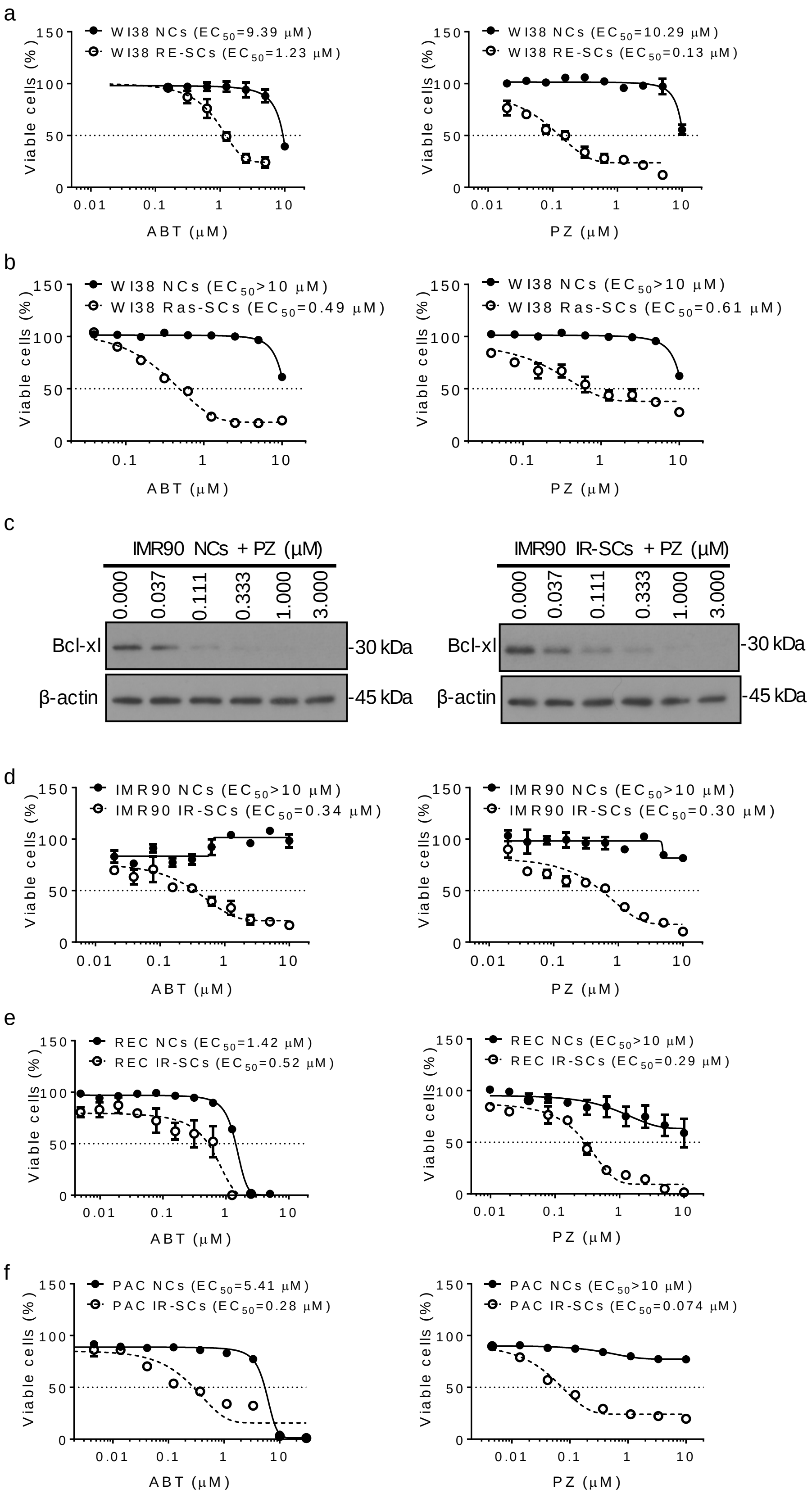
a
WI38 NCs (EC50=9.39 μM)
WI38 RE-SCs (EC50=1.23 μM)
WI38 NCs (EC50=10.29 μM)
WI38 RE-SCs (EC50=0.13 μM)
Viable cells (%)
ABT (μM)
PZ (μM)
b
WI38 NCs (EC50>10 μM)
WI38 Ras-SCs (EC50=0.49 μM)
WI38 NCs (EC50>10 μM)
WI38 Ras-SCs (EC50=0.61 μM)
c
IMR90 NCs + PZ (μM)
IMR90 IR-SCs + PZ (μM)
0.000
0.037
0.111
0.333
1.000
3.000
Bcl-xl
β-actin
-30 kDa
-45 kDa
d
IMR90 NCs (EC50>10 μM)
IMR90 IR-SCs (EC50=0.34 μM)
IMR90 NCs (EC50>10 μM)
IMR90 IR-SCs (EC50=0.30 μM)
e
REC NCs (EC50=1.42 μM)
REC IR-SCs (EC50=0.52 μM)
REC NCs (EC50>10 μM)
REC IR-SCs (EC50=0.29 μM)
f
PAC NCs (EC50=5.41 μM)
PAC IR-SCs (EC50=0.28 μM)
PAC NCs (EC50>10 μM)
PAC IR-SCs (EC50=0.074 μM)

Supplementary Figure 4. PZ is a potent and broad-spectrum senolytic agent. a-b. The percentage of viable cells in WI38 non-SCs (NCs), replicative senescent cells (RE-SCs) and *Ras* oncogene-induced senescent cells (Ras-SCs) after treatment with ABT263 (ABT) or PZ for 72 h. The data are presented as mean ± SEM of percentage of viable cells (n = 3 independent experiments). **c.** Western blot analysis shows that PZ induced Bcl-xl degradation in IMR90 NCs and IR-SCs in a dose-dependent manner after the cells were treated with PZ for 16 h. Similar results were got in at least two independent experiments. **d-f.** The percentage of viable cells in NCs and IR-SCs derived from IMR90 fibroblast cells (**d**), human renal epithelial cells (REC) (**e**) and preadipocytes (PAC) (**f**) after treatment with ABT or PZ for 72 h. The data are presented as mean ± SEM of percentage of viable cells (n = 3 independent experiments).

a

b

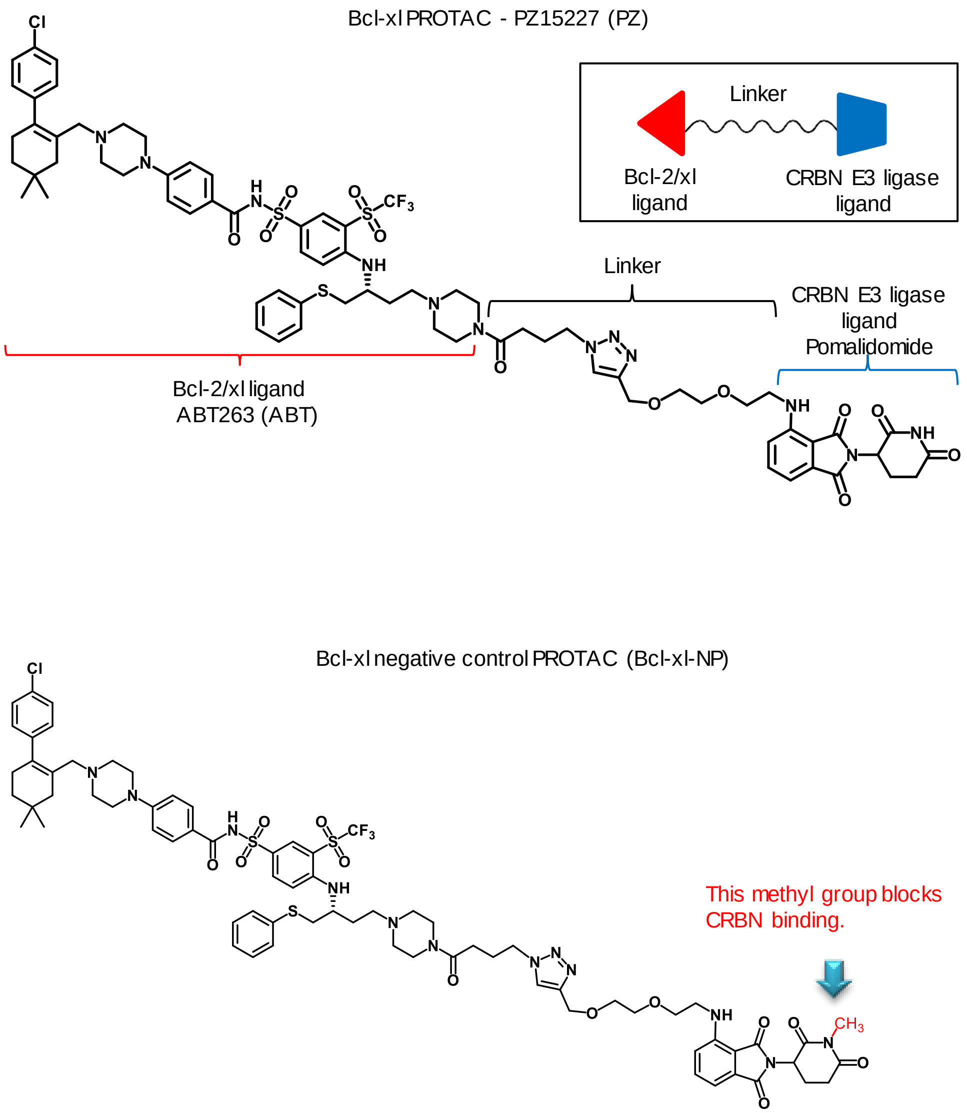

Supplementary Figure 5. Comparison of the structures of PZ and Bcl-xl-NP. The methyl group marked in red in the Bcl-xl-NP blocks CRBN binding.

a

PZ solubility & stability

Assays	PZ
Solubility (pH5.0)	< 0.06 μM
Solubility (pH7.4)	< 0.06 μM
Mouse plasma stability	$T_{1/2}$ > 500 min
Human plasma stability	$T_{1/2}$ > 500 min
Mouse liver microsomal stability	$T_{1/2}$ = 120 min
Human liver microsomal stability	$T_{1/2}$ = 139 min

b

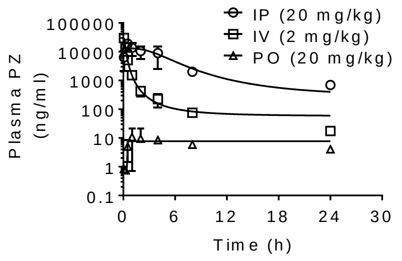

c

PZ mouse PK data

Male C57BL6 mice	IP (20 mg/kg)	IV (2 mg/kg)	PO (20 mg/kg)
Co/Cmax (ng/mL)	20967	44322	11.3
Tmax (h)	0.42	NA	2.0
$T_{1/2}$ (h)	7.1	5.8	ND
AUC0-t (ng.h/mL)	89567	13756	137
Tlast (h)	24.0	24.0	2.0-24.0
Cl (mL/min/kg)	NA	2.40	NA
Vss (L/kg)	NA	0.219	NA
F (%)	65.1	NA	0.1

d

PZ PLT toxicity in mice

Mice (n=3/group)	IP (mg/kg)	Platelet counts at 24 h (X 10^3/μl)
Vehicle	0	1333 (100)
PZ	20	1252 (94%)
ABT	20	331 (25%)

e

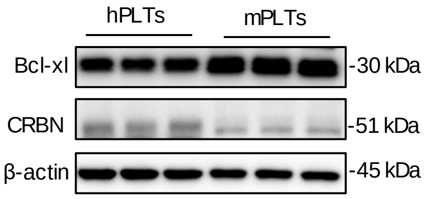

f

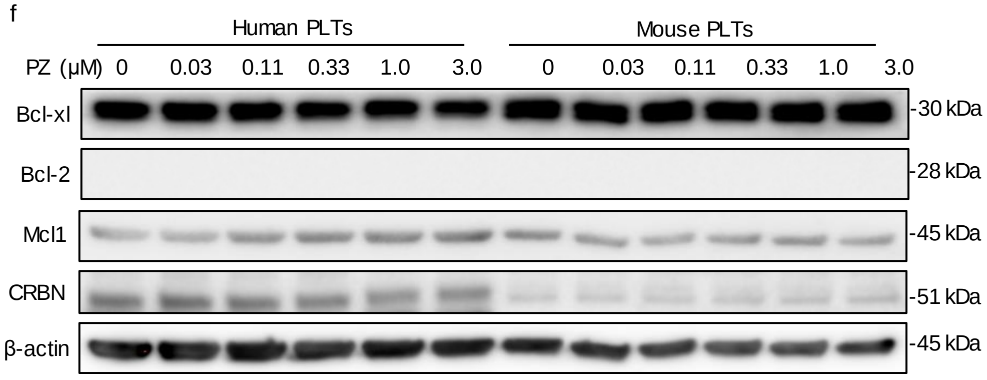

Supplementary Figure 6. Preliminary drug metabolism and pharmacokinetics (DMPK) and platelet toxicity studies in mice. a. Data from the analyses of PZ solubility and microsomal stability. **b-c.** Data from the preliminary mouse PK study. The data presented are mean ± SEM (n

= 3 mice for each dosing type). **d.** Preliminary platelet toxicity studies show that PZ is less toxic to platelets (PLTs) compared to ABT263 (ABT) in mice. **e.** Expression of Bcl-xl and CRBN in human and mouse PLTs analyzed by western blots. **f.** Western blot analyses showed that PZ had no significant effect on the levels of Bcl-xl, Bcl-2 or Mcl-1 in human or mouse PLTs after they were treated with different concentrations of PZ for 16 h. Similar results were got in at least two independent experiments, and a representative immunoblot is presented in **e** and **f**.

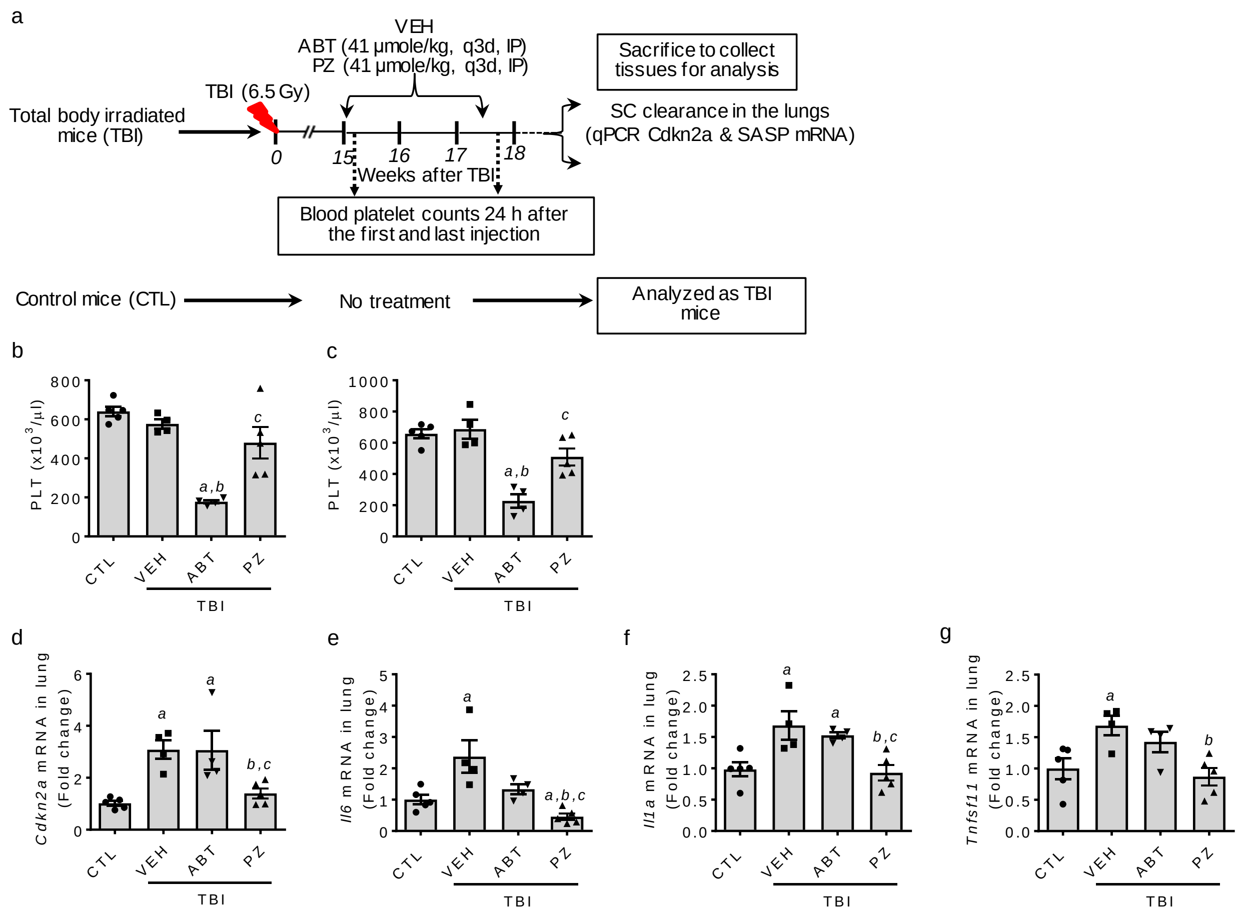

Supplementary Figure 7. PZ can effectively clear SCs in total body irradiated (TBI) mice without causing significant thrombocytopenia. a. Illustration of the experiment design. Fifteen weeks after exposure to 6.5 Gy TBI, TBI mice were given vehicle (VEH) or 41 μmol/kg of PZ or ABT263 (ABT) by IP injection every 3 days (q3d) for a total of 7 injections. Control non-irradiated and untreated mice (CTL) mice were analyzed as TBI mice. **b.** & **c.** Blood platelet counts in CTL and TBI mice were measured one day after receiving the 1st (**c**) and 7th (**d**) IP injection of VEH, ABT or PZ. **d-g.** Expression of *Cdkn2a* (**d**), *Il6* (**e**), *Il1a* (**f**) and *Tnfsf11* (**g**) mRNA in lung tissues from TBI mice treated with VEH, ABT or PZ was measured by quantitative PCR (qPCR). The data presented are mean ± SEM (n = 5, 4, 4, and 5 mice for CTL, and TBI + VEH, ABT and PZ, respectively). *a*, *b*, and *c*, $p < 0.05$ vs. CTL, TBI + VEH and TBI + ABT, respectively, determined by one-way ANOVA with Tukey's post-hoc tests, or Kruskal-Wallis one-way ANOVA with Dunn's post-hoc tests. The exact *P* values are provided in the Source Data file.

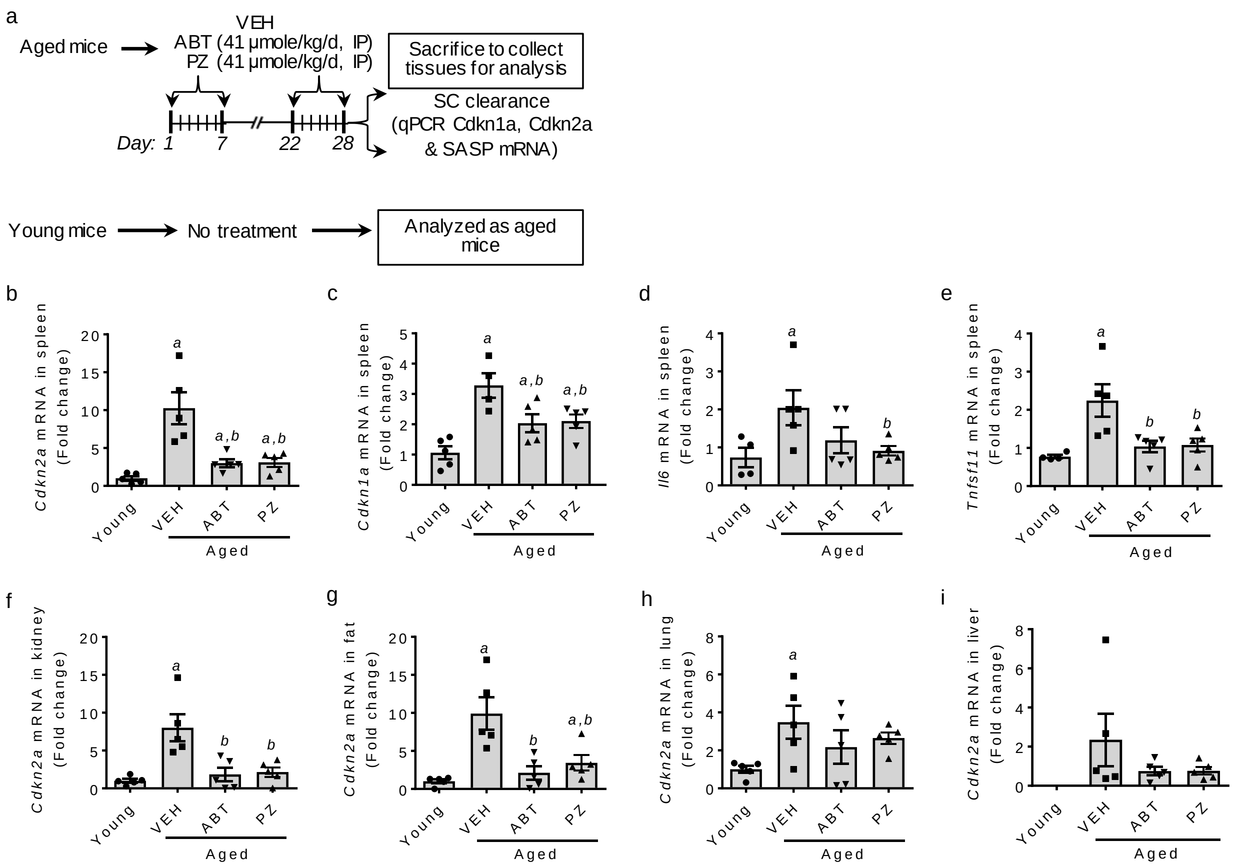

Supplementary Figure 8. A more intense dosing regimen of ABT263 (ABT) treatment can equally effective clear SCs as PZ in naturally aged mice. a. Illustration of the experiment design, which shows that mice were treated with two cycles of 7 daily IP injections of ABT or PZ. **b-i.** Expression of *Cdkn2a* (**b**), *Cdkn1a* (**c**), *Il6* (**d**), *Tnfsf11* (**e**) mRNA in the spleens, and expression of *Cdkn2a* mRNA in kidney (**f**), fat (**g**), lung (**h**) and liver (**i**) from young and naturally aged mice treated with VEH, ABT or PZ were measured by quantitative PCR. The data presented are mean ± SEM (n = 5 mice/group). *a* and *b*, $p < 0.05$ vs. Young and Aged + VEH, respectively, determined by one-way ANOVA with Tukey's post-hoc tests, or Kruskal-Wallis one-way ANOVA with Dunn's post-hoc tests. The exact *P* values are provided in the Source Data file.

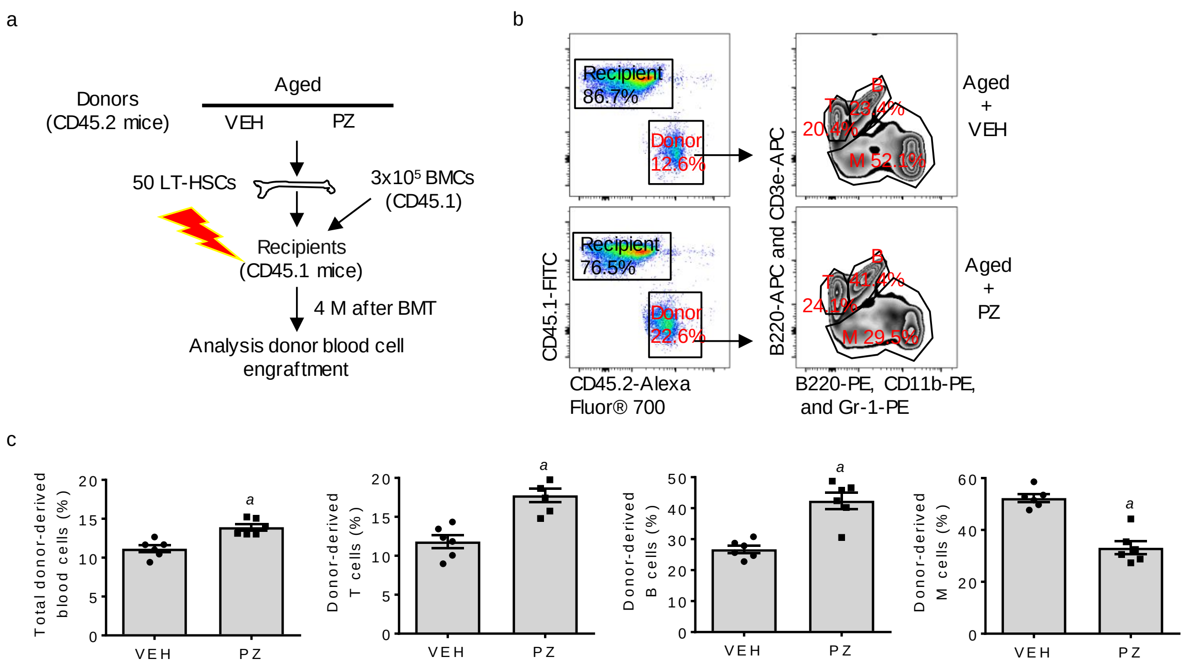

Supplementary Figure 9. PZ rejuvenates the function of HSCs in naturally aged mice. a. Illustration of the experiment design for the competitive repopulating assay (CRA). **b.** Representative flow cytometric analyses of donor-derived T cells, B cells and myeloid (M) cells in blood from recipients 4 months after receiving transplantation of HSCs from naturally aged mice treated with vehicle (VEH) or PZ as shown in **a**. **c.** Quantification of donor-derived total white blood cells ($CD45.2^+$), T cells (T; $CD45.2^+CD3e^+$), B cells (B; $CD45.2^+B220^+$) and myeloid cells (M; $CD45.2^+CD11b/Gr\text{-}1^+$) in the peripheral blood of recipient mice 4 months after HSC transplantation. The data presented are mean ± SEM (n = 6 mice/group). *a*, p=0.0012, 0.0006, 0.0003 and p<0.0001 vs. Aged + VEH for panel 1, 2, 3 and 4 from left to right in **c**, by two-tailed Student's *t* test.

Supplementary Table 1. Hematology profile in Young and naturally aged mice treated by vehicle (VEH), ABT263 (ABT) or PZ15227 (PZ)

			Young (n=8)		VEH (n=8)		ABT (n=6)		PZ (n=7)	
Parameters	Units	Reference range	Average	SEM	Average	SEM	Average	SEM	Average	SEM
WBC	$10^3/\mu L$	1.8-10.7	11.52	0.64	8.28	1.39	7.98	1.21	8.67	1.24
Neutrophil	$10^3/\mu L$	0.1-2.4	1.19	0.11	2.04	0.30	2.27	0.16	2.12	0.21
Lymphocyte	$10^3/\mu L$	0.9-9.3	9.55	0.57	5.81	1.13	4.94	1.00	5.61	0.97
Monocyte	$10^3/\mu L$	0.0-0.4	0.76	0.10	0.40	0.05	0.74	0.13	0.90	0.09
Eosinophil	$10^3/\mu L$	0.0-0.2	0.02	0.01	0.03	0.00	0.03	0.01	0.03	0.00
Basophil	$10^3/\mu L$	0.0-0.2	0.00	0.00	0.00	0.00	0.00	0.00	0.00	0.00
Neutrophil	%	6.6-38.9	10.51	1.02	26.35	2.44	30.70	3.33	25.65	2.00
Lymphocyte	%	55.8-91.6	82.79	0.81	67.80	2.69	59.26	3.62	63.05	2.53
Monocyte	%	0.0-7.5	6.51	0.60	5.40	0.57	9.56	1.27	10.90	0.96
Eosinophil	%	0.0-3.9	0.17	0.04	0.39	0.08	0.43	0.07	0.33	0.04
Basophil	%	0.0-0.2	0.03	0.01	0.07	0.02	0.05	0.02	0.05	0.01
RBC	$10^6/\mu L$	6.36-9.42	10.22	0.17	9.33	0.19	8.58	0.36	7.61	0.29
Hemoglobin	g/dL	11.0-15.1	12.84	0.14	11.70	0.18	10.53	0.44	9.37	0.47
Hematocrit	%	35.1-45.4	51.51	0.72	46.31	0.68	43.25	1.62	37.26	1.75
MCV	fL	45.4-60.3	50.44	0.52	49.70	0.55	50.48	0.76	48.86	0.76
MCH	pg	14.1-19.3	12.59	0.12	12.56	0.17	12.28	0.17	12.29	0.19
MCHC	g/dL	30.2-34.2	24.94	0.23	25.25	0.19	24.35	0.34	26.56	1.60
RDW	%	12.4-27.0	17.74	0.20	17.71	0.16	19.68	1.02	19.74	0.41
Platelet	$10^3/\mu L$	592-2972	628.88	29.77	814.88	72.29	155.67	21.29	636.43	55.45
MPV	fL	5.0-20.0	4.95	0.03	5.41	0.06	5.80	0.13	5.79	0.12

Note: WBC, white blood cell; RBC, red blood cell; MCV, mean corpuscular volume; MCH, mean cell hemoglobin; MCHC, mean corpuscular hemoglobin concentration; RDW, red cell distribution width

Supplementary Table 2. Suppliers for various compounds and cytokines

Compound name	Suppliers	Catalog #
ABT263	Selleckchem, Houston, TX,USA	S1001
MG132	Selleckchem, Houston, TX, USA	S2619
β-Mercaptoethanol	Sigma, St. Louis, MO, USA	M6250
Phosal 50 PG	American Lecithin Company, Oxford, CT, USA	368315-3130003/020
mSCF	Peprotech, Rocky Hill, NJ, USA	250-03
MIGLYOL® 810 N	IOI Oleochemical, Hamburg, Germany	-
Polysorbate 80	Spectrum Chemical, New Brunswick, NJ, USA	P0138
mIL-3	Peprotech, Rocky Hill, NJ, USA	213-13
mTPO	Peprotech, Rocky Hill, NJ, USA	315-14

Supplementary Table 3. Antibodies used for Western blotting analyses

Antibody	Clone	Antibody isotype	Catalog #	Concentration
Bcl-xl[1]	_	Rabbit IgG Polyclonal	2762S	1:1000
Mcl-1[1]	D35A5	Rabbit IgG Monoclonal	5453S	1:1000
Bcl-2[1]	50E3	Rabbit IgG Monoclonal	2870S	1:1000
β-actin[1]	13E5	Rabbit IgG Monoclonal	4970S	1:1000
CRBN[2]	_	Rabbit IgG Polyclonal	HPA045910	1:1000
UBA1[1]	_	Rabbit IgG Polyclonal	4890S	1:1000
SFT[4]	_	Rabbit IgG Polyclonal	ab176561	1:5000
Bcl-w[1]	31H4	Rabbit IgG Polyclonal	2724S	1:1000
Glutamine synthetase[3]	E-4	Mouse IgG Monoclonal	SC-74430	1:1000
CK1α[1]	_	Rabbit IgG Polyclonal	2655S	1:1000
IKZF1[1]	_	Rabbit IgG Polyclonal	5443S	1:1000
IKZF3[1]	D1C1E	Rabbit IgG Monoclonal	15103S	1:1000
Flag tag[1]	_	Rabbit IgG Polyclonal	2044S	1:1000
HA tag[1]	C29F4	Rabbit IgG Monoclonal	14031S	1:1000
β-actin[5]	C4	Mouse IgG Monoclonal	8691001	1:10000
Secondary antibody[1]	_	Anti-rabbit IgG	7074S	1:3500
Secondary antibody[1]	_	Anti-mouse IgG	7076S	1:5000

Footnotes: [1]Cell signaling, Danvers, MA, USA; [2]Sigma, St. Louis, MO, USA; [3]Santa Cruz Biotechnology, Santa Cruz, CA, USA; [4]Abcam, Cambridge, MA, USA; [5]MP Biomedicals, Santa Ana, CA, USA.

Supplementary Table 4. Taqman probes used for qRT-PCR

**Gene**	**Cat#**	**ID**
Human *Bcl2l1*	4351370	Hs00236329_m1
Human *GAPDH*	4351370	Hs02758991_g1
Human *CDKN2A*	4351370	Hs00923894_m1
Mouse *Cdkn2a*	4351370	Mm00494449_m1
Mouse *Hprt*	4351370	Mm01545399_m1
Mouse *Mrps2*	4351370	Mm00475528_m1
Mouse *Cdkn1a*	4351370	Mm00432448_m1
Mouse *Il6*	4351370	Mm00446190_m1
Mouse *Cxcl12*	4351370	Mm00445553_m1
Mouse *Mmp3*	4351370	Mm00440295_m1
Mouse *Tnfsf11*	4351370	Mm00441908_m1
Mouse *Il1a*	4351370	Mm99999060_ m1
Mouse *Mmp13*	4351370	Mm00439491_m1
Mouse *Cxcl12*	4351370	Mm00445553_m1

Footnotes: All probes were purchased from Thermo Fisher Scientific, Waltham, MA, USA.

Supplementary Table 5. Antibodies for flow cytometry and cell sorting

Antibody	Clone	Antibody	Conjugate	Catalog	Concentration
CD45R/B220[1]	RA3-6B2	IgG_{2a}	purified	553084	1:200
CD3e[1]	145-2C11	IgG_1	purified	553238	1:200
CD11b[1]	M1/70	IgG_{2b}	purified	553308	1:200
Gr-1[1]	RB6-8C5	IgG_{2b}	purified	553123	1:200
Ter-119[1]	Ter-119	IgG_{2b}	purified	553671	1:200
CD45R/B220[1]	RA3-6B2	IgG_{2a}	biotin	553086	1:200
CD3e[1]	145-2C11	IgG_1	biotin	553239	1:200
CD11b[1]	M1/70	IgG_{2b}	biotin	553309	1:200
Gr-1[1]	RB6-8C5	IgG_{2b}	biotin	553125	1:200
Ter-119[1]	Ter-119	IgG_{2b}	biotin	553672	1:200
CD45.1[1]	A20	IgG_{2a}	FITC	553775	1:200
CD45.2[1]	104	IgG_{2a}	Alexa Fluor® 700	560693	1:100
CD45R/B220[1]	RA3-6B2	IgG_{2a}	APC	553092	1:200
CD45R/B220[1]	RA3-6B2	IgG_{2a}	PE	553090	1:200
CD11b[1]	M1/70	IgG_{2a}	PE	553311	1:200
Gr-1[1]	RB6-8C5	IgG_{2a}	PE	561083	1:200
CD34[1]	RAM34	IgG_{2a}	Alexa Fluor® 700	560518	1:30
CD48[1]	HM48-1	IgG_1	BV421	562745	1:200
CD135(Flt3)[1]	A2F10	IgG_{2a}	PE	553842	1:200
CD127(IL7)[1]	A019D5	IgG_1	APC	564175	1:200
Streptavidin[1]	-	-	FITC	554060	1:100
Sca-1[1]	E13-161.7	IgG_{2a}	PE-Cy7	558162	1:200
c-kit[1]	2B8	IgG_{2b}	APC-Cy7	560185	1:200
CD150[3]	9D1	IgG_{2a}	BV785	115937	1:200
CD3e[3]	145-2C11	IgG	APC	553311	1:200
CD16/32[3]	93	IgG_{2a}	BV711	101337	1:200

Footnotes: [1]BD Biosciences, San Jose, CA; [2]eBioscience, San Jose, CA; [3]Biolegend, San Diego, CA.

Supplementary Note 1: Procedures for the Synthesis of PZ15227 (PZ) and Bcl-xl-NP

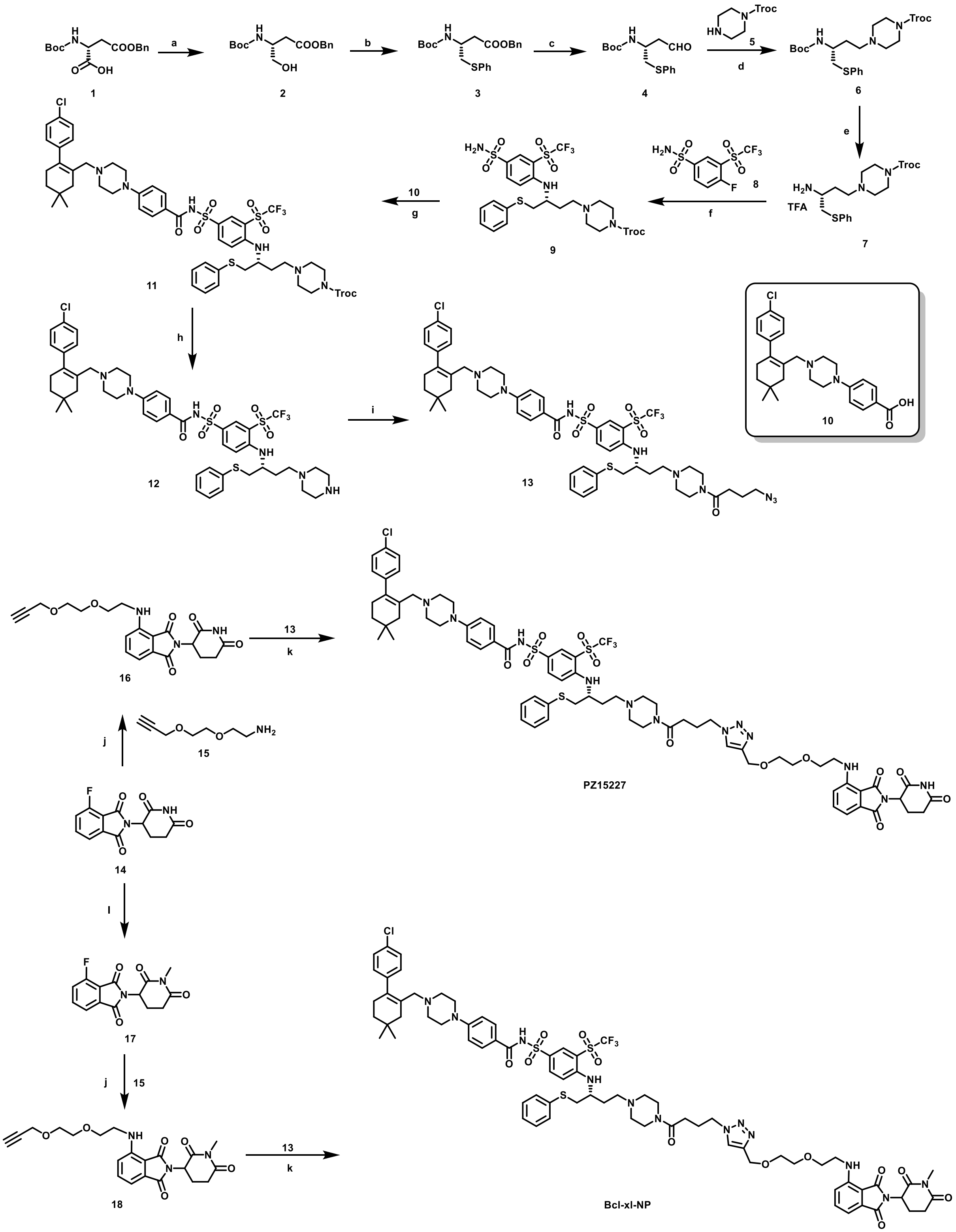

Scheme for the synthesis of PZ and Bcl-xl-NP. *Reagents and conditions:* (a) (1) *N*-methylmorpholine, isobutyl chloroformate, THF, -25 °C then -15 °C; (2) $NaBH_4$, THF, H_2O, -15 °C; (b) Bu_3P, diphenyl disulfide, toluene, 80 °C; (c) DIBAL-H, toluene, -78 °C; (d) compound **5**, $NaBH(OAc)_3$, TEA, DCM; (e) TFA, DCM; (f) compound **8**, TEA, acetonitrile, reflux; (g) compound **10**, EDCI, DMAP, DCM; (h) Zn, HOAc, THF; (i) 4-azidobutanoic acid, HATU, DIPEA, DCM; (j) compound **15**, DIPEA, DMF, 90 °C; (k) compound **13**, $CuSO_4{\cdot}5H_2O$, sodium L-ascorbate, ^tBuOH, THF, H_2O, 50 °C; (l) MeI, ^tBuOK, DMSO.

General Methods. THF, DCM, toluene, and acetonitrile were obtained via a solvent purification system by filtering through two columns packed with activated alumina and 4 Å molecular sieve, respectively. All other chemicals obtained from commercial sources were used without further purification. Flash chromatography was performed using silica gel (230–400 mesh) as the stationary phase. Reaction progress was monitored by thin layer chromatography (silica-coated glass plates) and visualized by UV light, and/or by LC–MS. NMR spectra were recorded in $CDCl_3$ at 400 MHz for ^1H NMR and 151 MHz for ^{13}C NMR. Chemical shifts δ are given in ppm using tetramethylsilane as an internal standard. Multiplicities of NMR signals are designated as singlet (s), broad singlet (*br* s), doublet (d), doublet of doublets (dd), triplet (t), quartet (q), and multiplet (m). All final compounds for biological testing were of ⩾98.0% purity as analyzed by LC–MS, performed on an Advion AVANT LC system with the expression CMS using a Thermo Accucore™ Vanquish™ C18+ UHPLC Column (1.5 µm, 50 x 2.1 mm) at 40 °C. Gradient elution was used for UHPLC with a mobile phase of acetonitrile and water containing 0.1% formic acid.

Synthesis of (*R*)-3-((*tert*-butoxycarbonyl)amino)-4-hydroxybutyric acid benzyl ester (2). *N*-Methylmorpholine (4.41 mL, 40.1 mmol) and isobutyl chloroformate (4.43 mL, 34.2 mmol) were added sequentially to a stirred solution of *N*-Boc-*D*-aspartic acid 4-benzyl ester (**1**) (10.0 g, 30.9 mmol) in THF (250 mL) at -25 °C. The resulting mixture was stirred at -25 °C for 30 min and allowed to warm to -15 °C. A solution of $NaBH_4$ (2.94 g, 77.7 mmol) in water (100 mL) was then added to the mixture in one portion (CAUTION: this procedure results in evolution of hydrogen gas). The mixture was stirred for 30 min at -15 °C and quenched with 1N HCl (aq.). The solution was extracted with ethyl acetate three times and the combined organic layers were

washed with brine, dried over Na_2SO_4, filtered, and evaporated to dryness under reduced pressure. The crude product was used directly in the next step. ^{1}H NMR (400 MHz, $CDCl_3$) δ 7.48–7.29 (m, 5H), 5.17 (*br* s, 1H), 5.12 (s, 2H), 4.05–3.93 (m, 1H), 3.69 (t, *J* = 5.2 Hz, 2H), 2.67 (d, *J* = 6.1 Hz, 2H), 2.38 (*br* s, 1H), 1.42 (s, 9H) ppm. LC-MS (ESI): *m/z* 310.3 $[M+H]^+$.

Synthesis of benzyl (*R*)-3-((*tert*-butoxycarbonyl)amino)-4-(phenylthio)butanoate (3). A mixture of (*R*)-3-((*tert*-butoxycarbonyl)amino)-4-hydroxybutyric acid benzyl ester (**2**) (30.9 mmol), diphenyl disulfide (8.8 g, 40.2 mmol), and Bu_3P (9.9 mL, 40.2 mmol) in toluene (150 mL) was heated at 80 °C under N_2 overnight. The mixture was cooled to room temperature and concentrated under reduced pressure. The crude product was purified by silica gel flash column chromatography using ethyl acetate and hexanes as eluents to afford the title compound (7.1 g, yield 57% in two steps). ^{1}H NMR (400 MHz, $CDCl_3$) δ 7.44–7.09 (m, 10H), 5.15 (*br* s, 1H), 5.08 (s, 2H), 4.24–3.97 (m, 1H), 3.23 (dd, *J* = 13.7, 5.5 Hz, 1H), 3.08 (dd, *J* = 13.6, 7.3 Hz, 1H), 2.80 (dd, *J* = 16.2, 5.1 Hz, 1H), 2.67 (dd, *J* = 16.4, 5.7 Hz, 1H), 1.40 (s, 9H) ppm. LC-MS (ESI): *m/z* 402.2 $[M+H]^+$.

Synthesis of *tert*-butyl *N*-[(2*R*)-4-oxo-1-(phenylsulfanyl)butan-2-yl]carbamate (4). DIBAL-H (1.2 M in toluene, 34.0 mL, 40.8 mmol) was added dropwise to a solution of compound **3** (7.1 g, 17.7 mmol) in toluene (80 mL) at -78 °C and stirred at the same temperature for 3 h. The reaction mixture was then quenched with NH_4Cl (aq.) and diluted with ethyl acetate. The resulting mixture was filtered and the filtrate was poured into water, and extracted with ethyl acetate. The combined organic phases were washed with brine, dried over Na_2SO_4, filtered, and evaporated to dryness under reduced pressure. The crude product was purified by silica gel flash column chromatography using ethyl acetate and hexanes as eluents to afford the title compound (4.16 g, yield 80%). LC-MS (ESI): *m/z* 296.2 $[M+H]^+$.

Synthesis of 2,2,2-trichloroethyl (*R*)-4-(3-((tert-butoxycarbonyl)amino)-4-(phenylthio)butyl)piperazine-1-carboxylate (6). To a mixture of compound **4** (592 mg, 2.00 mmol), compound **5** (753 mg, 2.88 mmol), and TEA (1.12 mL, 8.05 mmol) in DCM (15 mL) was added $NaBH(OAc)_3$ (638 mg, 3.00 mmol). The resulting solution was stirred at room temperature overnight before being poured into water and extracted with DCM. The combined organic phases were washed with brine, dried over Na_2SO_4, filtered, and evaporated to dryness under reduced pressure. The crude product was purified by silica gel flash column

chromatography using ethyl acetate and hexanes as eluents to afford the title compound (733 mg, yield 68%). ^{1}H NMR (400 MHz, $CDCl_3$) δ 7.43–7.36 (m, 2H), 7.32–7.27 (m, 2H), 7.19 (t, *J* = 7.3 Hz, 1H), 5.44 (*br* s, 1H), 4.76 (s, 2H), 3.99–3.84 (m, 1H), 3.72–3.49 (m, 4H), 3.23 (dd, *J* = 13.3, 4.6 Hz, 1H), 3.10–2.95 (m, 1H), 2.61–2.31 (m, 6H), 1.96–1.61 (m, 2H), 1.43 (s, 9H) ppm. LC-MS (ESI): *m/z* 540.1 $[M+H]^+$.

Synthesis of 2,2,2-trichloroethyl (*R*)-4-(3-amino-4-(phenylthio)butyl)piperazine-1-carboxylate TFA salt (7). To a solution of compound **6** (733 mg, 1.36 mmol) in DCM (5 mL) was added TFA (2.0 mL, 26.1 mmol). The mixture was stirred at room temperature for 1 h and solvent was removed under reduced pressure. The solid residue was washed with diethyl ether to afford the title compound (752 mg, yield 100%) as a white solid. ^{1}H NMR (400 MHz, $CDCl_3$): δ 7.41–7.33 (m, 2H), 7.31–7.26 (m, 2H), 7.23–7.15 (m, 1H), 4.74 (s, 2H), 3.73–3.41 (m, 4H), 3.20–2.66 (m, 5H), 2.58–2.28 (m, 6H), 1.84–1.57 (m, 2H) ppm. LC-MS (ESI): *m/z* 440.1 $[M+H]^+$.

Synthesis of 2,2,2-trichloroethyl (*R*)-4-(4-(phenylthio)-3-((4-sulfamoyl-2-((trifluoromethyl)sulfonyl)phenyl)amino)butyl)piperazine-1-carboxylate (9). A mixture of compound **7** (752 mg, 1.36 mmol), **8** (417 mg, 1.36 mmol), and TEA (945 μL, 6.80 mmol) in acetonitrile (20 mL) was stirred under reflux for 4 h. Solvent was evaporated under reduced pressure and the crude product was purified by silica gel flash column chromatography using ethyl acetate and hexanes as eluents to afford the title compound (780 mg, yield 79%) as a white solid. ^{1}H NMR (400 MHz, $CDCl_3$) δ 8.24 (d, *J* = 2.2 Hz, 1H), 7.84 (d, *J* = 9.1 Hz, 1H), 7.42–7.37 (m, 2H), 7.36–7.27 (m, 3H), 7.05 (d, *J* = 8.6 Hz, 1H), 6.65 (*br* s, 1H), 5.13 (*br* s, 2H), 4.76 (s, 2H), 4.02–3.88 (m, 1H), 3.75–3.40 (m, 4H), 3.16–2.97 (m, 2H), 2.82–2.26 (m, 6H), 2.19–2.05 (m, 1H), 1.85–1.77 (m, 1H) ppm. LC-MS (ESI): *m/z* 727.0 $[M+H]^+$.

Synthesis of 2,2,2-trichloroethyl (*R*)-4-(3-((4-(N-(4-(4-((4'-chloro-4,4-dimethyl-3,4,5,6-tetrahydro-[1,1'-biphenyl]-2-yl)methyl)piperazin-1-yl)benzoyl)sulfamoyl)-2-((trifluoromethyl)sulfonyl)phenyl)amino)-4-(phenylthio)butyl)piperazine-1-carboxylate (11). A mixture of compound **9** (780 mg, 1.07 mmol), **10** (470 mg, 1.07 mmol), EDCI (411 mg, 2.14 mmol), and DMAP (262 mg, 2.14 mmol) in DCM (40 mL) was stirred at room temperature overnight. Solvent was evaporated under reduced pressure and the crude product was purified by silica gel flash column chromatography using DCM and MeOH as eluents to afford the title

compound (859 mg, yield 70%) as a white solid. ^{1}H NMR (400 MHz, $CDCl_3$) δ 8.37 (d, *J* = 2.0 Hz, 1H), 8.13 (d, *J* = 9.2 Hz, 1H), 7.62 (d, *J* = 8.9 Hz, 2H), 7.41–7.27 (m, 6H), 7.12 (d, *J* = 8.7 Hz, 1H), 6.98 (d, *J* = 8.3 Hz, 2H), 6.79 (d, *J* = 9.0 Hz, 2H), 6.58 (d, *J* = 9.4 Hz, 1H), 4.75 (s, 2H), 3.95–3.81 (m, 1H), 3.63–3.38 (m, 4H), 3.33–3.23 (m, 4H), 3.11 (dd, *J* = 13.8, 4.9 Hz, 1H), 3.00 (dd, *J* = 13.9, 7.5 Hz, 1H), 2.81 (s, 2H), 2.46–2.01 (m, 15H), 1.75–1.65 (m, 1H), 1.46 (t, *J* = 6.3 Hz, 2H), 0.98 (s, 6H) ppm. LC-MS (ESI): *m/z* 1147.1 $[M+H]^+$.

Synthesis of (*R*)-4-(4-((4'-chloro-4,4-dimethyl-3,4,5,6-tetrahydro-[1,1'-biphenyl]-2-yl)methyl)piperazin-1-yl)-*N*-((4-((1-(phenylthio)-4-(piperazin-1-yl)butan-2-yl)amino)-3-((trifluoromethyl)sulfonyl)phenyl)sulfonyl)benzamide (12). Zinc powder (960 mg, 14.8 mmol) was added to a mixture of compound **11** (316 mg, 0.28 mmol) and AcOH (600 µL, 10.5 mmol) in THF (20 mL). The reaction mixture was stirred at room temperature for 5 h. The solid was removed by filtration and the filtrate was poured into water and extracted with ethyl acetate. The combined organic phases were washed with brine, dried over Na_2SO_4, filtered, and evaporated to dryness under reduced pressure. The crude product was purified by silica gel flash column chromatography using DCM, MeOH, and TEA as eluents to afford compound **12** (210 mg, yield 78%). ^{1}H NMR (400 MHz, $CDCl_3$) δ 8.21 (s, 1H), 7.93 (d, *J* = 9.2 Hz, 1H), 7.85 (d, *J* = 8.6 Hz, 2H), 7.33–7.24 (m, 2H), 7.22–7.08 (m, 5H), 6.92 (d, *J* = 8.3 Hz, 2H), 6.77 (d, *J* = 8.4 Hz, 1H), 6.66 (d, *J* = 8.7 Hz, 2H), 6.46 (d, *J* = 9.3 Hz, 1H), 3.83–3.67 (m, 1H), 3.17–3.08 (m, 4H), 3.02–2.92 (m, 5H), 2.89–2.78 (m, 1H), 2.72 (s, 2H), 2.64–2.13 (m, 12H), 2.04–1.91 (m, 3H), 1.62–1.49 (m, 1H), 1.39 (t, *J* = 6.3 Hz, 2H), 0.91 (s, 6H) ppm. LC-MS (ESI): *m/z* 973.2 $[M+H]^+$.

Synthesis of (*R*)-*N*-((4-((4-(4-(4-azidobutanoyl)piperazin-1-yl)-1-(phenylthio)butan-2-yl)amino)-3-((trifluoromethyl)sulfonyl)phenyl)sulfonyl)-4-(4-((4'-chloro-4,4-dimethyl-3,4,5,6-tetrahydro-[1,1'-biphenyl]-2-yl)methyl)piperazin-1-yl)benzamide (13). HATU (30 mg, 0.079 mmol) was added to a mixture of compound **12** (50 mg, 0.051 mmol), 4-azidobutanoic acid (6.7 mg, 0.052 mmol), DIPEA (13.5 µL, 0.082 mmol) in 2 mL DCM. The mixture was stirred at room temperature for 1 h. The solvent was removed under reduced pressure and the crude product was purified via column chromatography using DCM and methanol as eluents to afford the title compound (40 mg, yield 72%). ^{1}H NMR (400 MHz, $CDCl_3$) δ 8.35 (d, *J* = 2.2 Hz, 1H), 8.11 (dd, *J* = 9.2, 2.2 Hz, 1H), 7.67 (d, *J* = 8.9 Hz, 2H), 7.40–7.35 (m, 2H), 7.34–7.27 (m,

3H), 7.26–7.24 (m, 2H), 7.09 (d, *J* = 8.5 Hz, 1H), 7.02–6.96 (m, 2H), 6.76 (d, *J* = 9.0 Hz, 2H), 6.58 (d, *J* = 9.4 Hz, 1H), 3.99–3.81 (m, 1H), 3.72–3.60 (m, 1H), 3.53–3.33 (m, 5H), 3.32–3.22 (m, 4H), 3.11 (dd, *J* = 13.8, 4.9 Hz, 1H), 3.00 (dd, *J* = 13.8, 7.5 Hz, 1H), 2.87 (s, 2H), 2.51–2.20 (m, 14H), 2.19–2.08 (m, 1H), 2.06–1.99 (m, 2H), 1.97–1.85 (m, 2H), 1.71–1.64 (m, 1H), 1.46 (t, *J* = 6.4 Hz, 2H), 0.97 (s, 6H) ppm. LC-MS (ESI): *m/z* 1084.4 $[M+H]^+$.

Synthesis of 2-(2,6-dioxopiperidin-3-yl)-4-((2-(2-(prop-2-yn-1-yloxy)ethoxy)ethyl)amino)isoindoline-1,3-dione (16): Compound **14** (107 mg, 0.39 mmol), amine **15** (84 mg, 0.58 mmol), and DIPEA (193 μL, 1.17 mmol) in DMF (5 mL) were stirred at 90 °C for 16 h. The reaction mixture was poured into water and extracted with ethyl acetate. The organic phase was washed with water, brine, dried over Na_2SO_4, filtered, and evaporated to dryness. The resulting mixture was purified by silica gel flash column chromatography using ethyl acetate and hexanes as eluents to afford the title compound (50 mg, yield 32%) as a green solid. ^1H NMR (400 MHz, $CDCl_3$) δ 7.98 (s, 1H), 7.62–7.35 (m, 1H), 7.11 (d, *J* = 7.1 Hz, 1H), 6.93 (d, *J* = 8.5 Hz, 1H), 4.92 (dd, *J* = 11.9, 5.3 Hz, 1H), 4.21 (d, *J* = 2.3 Hz, 2H), 3.78–3.66 (m, 6H), 3.49 (t, *J* = 5.4 Hz, 2H), 2.93–2.68 (m, 3H), 2.48–2.41 (m, 1H), 2.18–2.09 (m, 1H) ppm. LC-MS (ESI): *m/z* 400.0 $[M+H]^+$.

Synthesis of 4-fluoro-2-(1-methyl-2,6-dioxopiperidin-3-yl)isoindoline-1,3-dione (17): A mixture of **14** (100 mg, 0.36 mmol), MeI (25 μL, 0.40 mmol), and ^{t}BuOK (81 mg, 0.72 mmol) in DMSO (1.5 mL) was stirred at room temperature for 2 h. The reaction was then quenched by adding water and extracted with ethyl acetate. The organic phase was washed with brine, dried over Na_2SO_4, filtered, and evaporated to dryness. The crude product was purified by silica gel flash column chromatography to afford the title compound (47 mg, yield 45%). ^1H NMR (400 MHz, $CDCl_3$) δ 7.82–7.68 (m, 2H), 7.46–7.39 (m, 1H), 5.03–4.93 (m, 1H), 3.21 (s, 3H), 3.04–2.94 (m, 1H), 2.93–2.71 (m, 2H), 2.18–2.06 (m, 1H) ppm. LC-MS (ESI): *m/z* 291.1 $[M+H]^+$.

Synthesis of 2-(1-methyl-2,6-dioxopiperidin-3-yl)-4-((2-(2-(2-(prop-2-yn-1-yloxy)ethoxy)ethoxy)ethyl)amino)isoindoline-1,3-dione (18): A mixture of **17** (50 mg, 0.17 mmol), **15** (25 mg, 0.17 mmol), and DIPEA (145 μL, 0.88 mmol) in DMF (1.5 mL) was stirred at 90 °C overnight. Water (10 mL) was added and the resulting mixture was extracted with ethyl acetate. The organic phase was washed with brine, dried over Na_2SO_4, filtered, and evaporated to dryness. The crude product was purified by column chromatography to afford the title compound

(34 mg, yield 48%). ^{1}H NMR (400 MHz, $CDCl_3$) δ 7.49 (dd, *J* = 8.5, 7.1 Hz, 1H), 7.10 (d, *J* = 7.1 Hz, 1H), 6.93 (d, *J* = 8.6 Hz, 1H), 6.48 (t, *J* = 5.8 Hz, 1H), 5.01–4.81 (m, 1H), 4.21 (d, *J* = 2.4 Hz, 2H), 3.79–3.62 (m, 6H), 3.57–3.42 (m, 2H), 3.21 (s, 3H), 3.06–2.65 (m, 3H), 2.44 (t, *J* = 2.4 Hz, 1H), 2.18–2.06 (m, 1H) ppm. LC-MS (ESI): *m/z* 414.1 $[M+H]^+$.

Synthesis of 4-(4-((4'-chloro-4,4-dimethyl-3,4,5,6-tetrahydro-[1,1'-biphenyl]-2-yl)methyl)piperazin-1-yl)-N-((4-(((2R)-4-(4-(4-(4-((2-(2-((2-(2,6-dioxopiperidin-3-yl)-1,3-dioxoisoindolin-4-yl)amino)ethoxy)ethoxy)methyl)-1H-1,2,3-triazol-1-yl)butanoyl)piperazin-1-yl)-1-(phenylthio)butan-2-yl)amino)-3-((trifluoromethyl)sulfonyl)phenyl)sulfonyl)benzamide (PZ15227): To a mixture of compound **13** (25.0 mg, 0.023 mmol), compound **16** (11.0 mg, 0.028 mmol) in *t*-BuOH-THF (1:2, v/v, 3 mL) under Argon was added $CuSO_4·5H_2O$ (1.15 mg, 0.0046 mmol) and sodium L-ascorbate (0.91 mg, 0.0046 mmol) in water (0.3 mL). The mixture was stirred at 50 °C overnight, cooled to room temperature, and extracted with DCM. The organic phase was washed with brine, dried over Na_2SO_4, filtered, and evaporated to dryness. The crude product was purified by silica gel flash column chromatography using DCM and methanol as eluents to afford the title compound (23 mg, yield 67%). ^{1}H NMR (400 MHz, $CDCl_3$) δ 9.05 (*br* s, 1H), 8.36 (s, 1H), 8.10 (d, *J* = 7.8 Hz, 1H), 7.79–7.64 (m, 3H), 7.54–7.42 (m, 1H), 7.43–7.22 (m, 7H), 7.10–7.02 (m, 2H), 6.99 (d, *J* = 7.2 Hz, 2H), 6.92 (d, *J* = 8.8 Hz, 1H), 6.77 (d, *J* = 8.8 Hz, 2H), 6.61 (d, *J* = 9.3 Hz, 1H), 6.50 (*br* s, 1H), 4.99–4.85 (m, 1H), 4.69 (s, 2H), 4.42–4.37 (m, 2H), 4.00–3.77 (m, 1H), 3.80–3.58 (m, 8H), 3.52– 3.20 (m, 8H), 3.12–3.00 (m, 2H), 2.84–2.75 (m, 5H), 2.45–1.98 (m, 20H) , 1.74–1.60 (m, 1H), 1.46 (t, *J* = 6.4 Hz, 2H), 0.97 (s, 6H) ppm. ^{13}C NMR (151 MHz, $CDCl_3$) δ 171.5, 170.0, 170.0, 169.3, 168.9, 168.9, 167.7, 167.7, 164.9, 154.2, 151.8, 146.8, 145.1, 141.7, 137.9, 136.0, 135.4, 135.3, 134.5, 132.5, 132.1, 131.1, 129.8, 129.7, 129.3, 128.8, 128.3, 127.5, 127.0, 123.3, 123.1, 121.1, 120.3, 119.0, 116.8, 113.4, 112.9, 111.6, 110.3, 108.7, 70.6, 69.8, 69.4, 64.7, 60.4, 53.8, 53.2, 52.5, 52.3, 50.7, 49.5, 49.0, 46.9, 45.1, 42.4, 41.5, 41.4, 39.0, 35.6, 31.5, 30.9, 30.3, 29.1, 28.9, 28.1, 25.5, 22.8. LC-MS (ESI): *m/z* 1483.4 $[M+H]^+$.

Synthesis of 4-(4-((4'-chloro-4,4-dimethyl-3,4,5,6-tetrahydro-[1,1'-biphenyl]-2-yl)methyl)piperazin-1-yl)-N-((4-(((2R)-4-(4-(4-(4-((2-(2-((2-(1-methyl-2,6-dioxopiperidin-3-yl)-1,3-dioxoisoindolin-4-yl)amino)ethoxy)ethoxy)methyl)-1H-1,2,3-triazol-1-yl)butanoyl)piperazin-1-yl)-1-(phenylthio)butan-2-yl)amino)-3-((trifluoromethyl)sulfonyl)phenyl)sulfonyl)benzamide (Bcl-xl-NP) : To a mixture of

compound **13** (30.0 mg, 0.028 mmol), compound **18** (12.6 mg, 0.030 mmol) in *t*-BuOH-THF (1:2, v/v, 3 mL) under Argon was added $CuSO_4 \cdot 5H_2O$ (1.4 mg, 0.0056 mmol) and sodium L-ascorbate (1.1 mg, 0.0056 mmol) in water (0.3 mL). The mixture was stirred at 50 °C overnight, cooled to room temperature, and extracted with DCM. The organic phase was washed with brine, dried over Na_2SO_4, filtered, and evaporated to dryness. The crude product was purified by silica gel flash column chromatography using DCM and methanol as eluents to afford the title compound. (31 mg, yield 74%). ^{1}H NMR (400 MHz, $CDCl_3$) δ 8.36 (s, 1H), 8.10 (d, *J* = 9.2 Hz, 1H), 7.74–7.64 (m, 2H), 7.62 (s, 1H), 7.47 (dd, *J* = 8.5, 7.1 Hz, 1H), 7.41–7.27 (m, 5H), 7.26–7.22 (m, 2H), 7.07 (d, *J* = 7.1 Hz, 2H), 7.02–6.96 (m, 2H), 6.91 (d, *J* = 8.5 Hz, 1H), 6.75 (d, *J* = 8.6 Hz, 2H), 6.62 (d, *J* = 9.4 Hz, 1H), 6.48 (t, *J* = 5.7 Hz, 1H), 4.92 (dd, *J* = 12.2, 5.6 Hz, 1H), 4.69 (s, 2H), 4.41 (t, *J* = 6.4 Hz, 2H), 3.97–3.84 (m, 1H), 3.73–3.65 (m, 6H), 3.53–3.22 (m, 10H), 3.19 (s, 3H), 3.11 (dd, *J* = 13.8, 5.0 Hz, 1H), 3.04–2.93 (m, 2H), 2.90–2.68 (m, 4H), 2.43–1.99 (m, 20H), 1.72–1.61 (m, 1H), 1.46 (t, *J* = 6.7 Hz, 2H), 0.97 (s, 6H) ppm. ^{13}C NMR (151 MHz, $CDCl_3$) δ 171.4, 169.7, 169.5, 169.2, 167.8, 165.0, 154.1, 151.7, 146.8, 145.3, 141.7, 137.9, 136.0, 135.6, 135.2, 134.5, 132.5, 132.2, 131.1, 129.8, 129.7, 129.3, 128.7, 128.4, 127.5, 127.1, 123.3, 123.0, 121.2, 120.4, 119.0, 116.8, 113.5, 112.9, 111.6, 110.3, 108.7, 70.6, 69.9, 69.6, 64.6, 60.5, 53.8, 53.3, 52.6, 52.3, 50.7, 49.6, 49.6, 46.9, 45.1, 42.4, 41.4, 41.4, 39.0, 35.6, 31.9, 30.9, 30.2, 29.1, 29.0, 28.1, 27.3, 25.5, 22.1 ppm. LC-MS (ESI): *m/z* 1497.7 $[M+H]^+$.
